# Quantitative Analysis of the *Drosophila* Segmentation Regulatory Network Using Pattern Generating Potentials

**DOI:** 10.1371/journal.pbio.1000456

**Published:** 2010-08-17

**Authors:** Majid Kazemian, Charles Blatti, Adam Richards, Michael McCutchan, Noriko Wakabayashi-Ito, Ann S. Hammonds, Susan E. Celniker, Sudhir Kumar, Scot A. Wolfe, Michael H. Brodsky, Saurabh Sinha

**Affiliations:** 1 Department of Computer Science, University of Illinois at Urbana-Champaign, Urbana-Champaign, Illinois, United States of America; 2 Program in Gene Function and Expression, University of Massachusetts Medical School, Worcester, Massachusetts, United States of America; 3 Department of Molecular Medicine, University of Massachusetts Medical School, Worcester, Massachusetts, United States of America; 4 Center for Evolutionary Functional Genomics, Biodesign Institute, Arizona State University, Tempe, Arizona, United States of America; 5 Department of Genome Dynamics, Berkeley Drosophila Genome Project, Lawrence Berkeley National Laboratory, Berkeley, California, United States of America; 6 Department of Biochemistry and Molecular Pharmacology, University of Massachusetts Medical School, Worcester, Massachusetts, United States of America; 7 Institute of Genomic Biology, University of Illinois at Urbana-Champaign, Urbana-Champaign, Illinois, United States of America; University of California Berkeley, United States of America

## Abstract

*Cis*-regulatory modules that drive precise spatial-temporal patterns of gene expression are central to the process of metazoan development. We describe a new computational strategy to annotate genomic sequences based on their “pattern generating potential” and to produce quantitative descriptions of transcriptional regulatory networks at the level of individual protein-module interactions. We use this approach to convert the qualitative understanding of interactions that regulate *Drosophila* segmentation into a network model in which a confidence value is associated with each transcription factor-module interaction. Sequence information from multiple *Drosophila* species is integrated with transcription factor binding specificities to determine conserved binding site frequencies across the genome. These binding site profiles are combined with transcription factor expression information to create a model to predict module activity patterns. This model is used to scan genomic sequences for the potential to generate all or part of the expression pattern of a nearby gene, obtained from available gene expression databases. Interactions between individual transcription factors and modules are inferred by a statistical method to quantify a factor's contribution to the module's pattern generating potential. We use these pattern generating potentials to systematically describe the location and function of known and novel *cis*-regulatory modules in the segmentation network, identifying many examples of modules predicted to have overlapping expression activities. Surprisingly, conserved transcription factor binding site frequencies were as effective as experimental measurements of occupancy in predicting module expression patterns or factor-module interactions. Thus, unlike previous module prediction methods, this method predicts not only the location of modules but also their spatial activity pattern and the factors that directly determine this pattern. As databases of transcription factor specificities and in vivo gene expression patterns grow, analysis of pattern generating potentials provides a general method to decode transcriptional regulatory sequences and networks.

## Abstract 2

A new computational method uses gene expression databases and transcription factor binding specificities to describe regulatory elements in the *Drosophila* A/P patterning network in unprecedented detail.

## Author Summary

The developmental program specifying segmentation along the anterior-posterior axis of the *Drosophila* embryo is one of the best studied examples of transcriptional regulatory networks. Previous work has identified the location and function of dozens of DNA segments called *cis*-regulatory “modules” that regulate several genes in precise spatial patterns in the early embryo. In many cases, transcription factors that interact with such modules have also been identified. We present a novel computational framework that turns a qualitative and fragmented understanding of modules and factor-module interactions into a quantitative, systems-level view. The formalism utilizes experimentally characterized binding specificities of transcription factors and gene expression patterns to describe how multiple transcription factors (working as activators or repressors) act together in a module to determine its regulatory activity. This formalism can explain the expression patterns of known modules, infer factor-module interactions and quantify the potential of an arbitrary DNA segment to drive a gene's expression. We have also employed databases of gene expression patterns to find novel modules of the regulatory network. As databases of binding motifs and gene expression patterns grow, this new approach provides a general method to decode transcriptional regulatory sequences and networks.

## Introduction

A central challenge in understanding metazoan genome sequences is to identify and annotate genomic regions that regulate the complex spatial and temporal patterns of gene transcription. Analysis of the regulatory regions for many individual genes has typically identified discrete enhancers or “*cis*-regulatory modules” (CRMs) that are approximately 1 Kbp long and located at distances ranging from immediately adjacent to the start of transcription to 100 Kbp away. These CRMs are composed of transcription factor binding sites that integrate information about the concentration of relevant factors to determine the quantitative contribution of each CRM to the expression of its target gene [1]. A variety of experimental approaches has been utilized to identify and characterize CRMs in single gene or genome-wide studies. For example, approximately 50 CRMs involved in the anterior-posterior (A/P) segmentation of the blastoderm stage *Drosophila* embryo [2] have been identified by reporter gene assays. A combination of genetic studies, CRM mutagenesis, and DNA binding assays has identified individual transcription factors (TFs) that influence the activity of these modules.

Genome-wide identification of TF binding loci has been carried out using chromatin immunoprecipitation (ChIP) in a variety of systems, including yeast and cultured cells [3],[4]. ChIP of TFs that act to regulate dorsal-ventral or anterior-posterior patterning in *Drosophila* embryos identifies a set of bound genomic regions that is highly enriched in functional targets but also includes many regions whose contribution to patterned gene expression is currently unclear [5]–[8]. Furthermore, while ChIP can identify targets in specific stages or cell types, a clear technical challenge for ChIP-based methods is how to systematically characterize the genome-wide occupancy of the large number of TFs in metazoans across the vast number of distinct expression states that occur during developmental and physiological processes.

Computational analysis provides a complementary means to discover functional TF–CRM interactions in the genome. Past attempts to identify CRMs often searched for clusters of putative binding sites for combinations of TFs that act in common biological processes [9] and have been particularly successful in the identification of *Drosophila* segmentation modules [10]. The statistical power of these approaches is increased by filtering for evolutionary conservation of either individual sites or regions with clusters of sites [11]–[13]. In parallel, new methods to systematically determine TF-DNA binding specificities [14],[15] have the potential to generate a relatively large number of binding specificities (“motifs”) in a short time. Spurred by these advances and the increasing availability of new genomic sequences, computational approaches could, in principle, be applied more globally to determine the transcriptional regulatory function of genomic sequences. However, several problems complicate the global computational annotation of CRMs and TF–CRM interactions. First, there is the problem of overlapping specificities; many TFs, particularly those in common structural families such as homeodomains, have highly similar DNA binding specificities [16], making it difficult to assign conserved binding sites to an individual TF. Second, there is the problem of selecting the optimal combinations of TFs that should be tested together for clusters of sites; this becomes increasingly difficult as more expression states are considered. Third, there is the problem of TF pleiotropy; for example, a subset of TFs expressed during segmentation of the *Drosophila* blastoderm act again during cell fate specification in the nervous system. Genomic segments with overrepresentation of binding sites for these TFs might act during either developmental stage. A related problem is the identification of CRMs for genes with multiple expression domains; cluster-based analysis does not automatically attribute a specific expression domain to each CRM. Finally, there is the challenge of evaluating the relevance of individual TF–CRM interactions; while combining binding site scores for multiple TFs increases the sensitivity of CRM detection, the contribution of any individual TF to CRM function is typically smaller and more difficult to associate with a significance value.

We describe a new approach for CRM identification and annotation that begins to address these issues. It employs a new method to estimate the potential of any genomic segment to drive a spatial expression pattern matching that of its nearby gene. This “pattern generating potential” is computed by combining information from experimentally determined TF binding motifs, TF expression patterns, and a comprehensive database of in situ gene expression images of the *Drosophila* embryo. For this approach, we developed an efficiently computable, regression-based model of expression patterns as a function of evolutionarily conserved binding sites, with parameters learned from a collection of experimentally characterized CRMs. By incorporating TF expression patterns into the model, the contribution of potential binding sites for a factor are only considered in the subset of cells that express that factor. Genomic regions are annotated as potential CRMs based on functional combinations of TF binding sites, while rejecting clusters of overrepresented binding sites that are inconsistent with the relevant gene expression pattern. Whether an individual CRM contributes to all or part of the expression pattern is an automatic result of the method. The contribution of any individual TF to this pattern can be quantitatively evaluated by examining the effect of disrupting the TF's expression pattern on the predicted activity of the CRM. We use this method to annotate genomic sequences with the potential to regulate the initial stages of segmentation in the *Drosophila* embryo. We exploit this approach to produce an associated transcriptional regulatory network model in which each TF–CRM interaction is associated with a confidence value. We demonstrate that this approach provides additional insights into how multiple CRMs contribute to expression patterns and how individual TFs can directly or indirectly regulate the expression of multiple target genes. This study represents a generalizable approach to produce predictive models of genome function and regulatory networks.

## Results

### Cross-Species Comparison Dramatically Improves Prediction of TF Occupancy

The availability of genome sequences for multiple *Drosophila* species provides an opportunity to optimize quantitative modeling of functional TF occupancy along the genome. The basic assumption of this approach is that CRMs with conserved activity across these species will maintain some binding activity for each requisite TF while binding sites in non-functional regions will be less conserved. We used genome-wide profiles of binding motif scores for 10 TFs (BCD, CAD, HB, KNI, KR, GT, HKB, TLL, FKH, and CIC) involved in the initial stages of anterior-posterior patterning or segmentation in the embryo. These profiles were generated using the Hidden Markov Model–based Stubb program [17] that captures both weak and strong motif matches in a probabilistic framework. We combined the motif profiles from *D. melanogaster* and 10 other *Drosophila* genomes [13], by averaging scores from orthologous ∼500 bp regions, to create a multi-species motif profile that incorporates evolutionary conservation. Because species more closely related to *D. melanogaster* are better represented in the currently sequenced set of genomes, this phylogenetic comparison is weighted more heavily towards *D. melanogaster* than more distant species. In an alternative approach designed to reflect the evolutionary distances among the sequenced species, we modeled the motif score of a region as a random variable evolving through Brownian Motion dynamics along the branches of the evolutionary tree [18], and computed the expected tree-wide average of this variable given its observed values in the extant species (Methods). This computation is performed using a new “upward-downward” algorithm that scales linearly with the number of species. These single and multi-species motif profiles are made available through the “Genome Surveyor” interface [14] at http://veda.cs.uiuc.edu/lmcrm/.

We used published ChIP-on-chip data for eight TFs (BCD, CAD, GT, HB, KNI, KR, HKB, and TLL) [6],[19] to compare the ability of different motif profiles to distinguish the top 100 bound regions from a random set of non-coding regions (Methods). As Table 1 reveals, single species motif scores show significant discrimination between bound and random sequences (*p* value <0.01) for each TF, with especially strong discrimination in the cases of BCD and HKB (*p* value = 2.0E-25 and 5.7E-23, respectively). We find a dramatic improvement in this discriminative ability when using multi-species motif profiles (e.g., the *p* value improves from 1.5E-5 to 1.9E-27 for CAD, from 1.8E-3 to 7.0E-15 for HB, and from 2.0E-4 to 3.1E-20 for TLL). The two schemes for combining multi-species profiles produce comparable results by this measure, which are significantly better than results produced by corresponding two-species (*D. mel.* and *D. pse.*) motif profiles. Both multi-species methods were also tested in CRM predictions below.

**Table 1:** Agreement of motif profiles with ChIP-on-chip data.

TF	Single Species	2 Species (Simple Averaging)	11 Species (Simple Averaging)	11 Species (Brownian Motion-Based Averaging)
BCD	2.0E-25	2.0E-33	2.0E-45	1.6E-45
CAD	1.5E-5	1.5E-15	5.5E-27	1.9E-27
GT	8.3E-7	8.5E-11	2.7E-21	2.3E-22
HB	1.8E-3	3.3E-5	5.5E-14	7.0E-15
KNI	1.8E-4	2.3E-5	1.1E-9	7.5E-10
KR	2.6E-13	3.5E-17	4.2E-29	3.6E-31
HKB	5.7E-23	8.7E-23	1.5E-35	7.4E-35
TLL	2.0E-4	1.3E-8	1.9E-17	3.1E-20

Shown for each transcription factor (TF), and each motif scoring scheme (columns 2–5), is the *p* value of discrimination between ChIP peaks of that TF and random non-coding sequences, using the motif scores of those sequences and the Wilcoxon Rank-Sum test.

### Prediction of Expression Patterns for Known CRMs by Generalized Linear Regression

**Figure 1:**
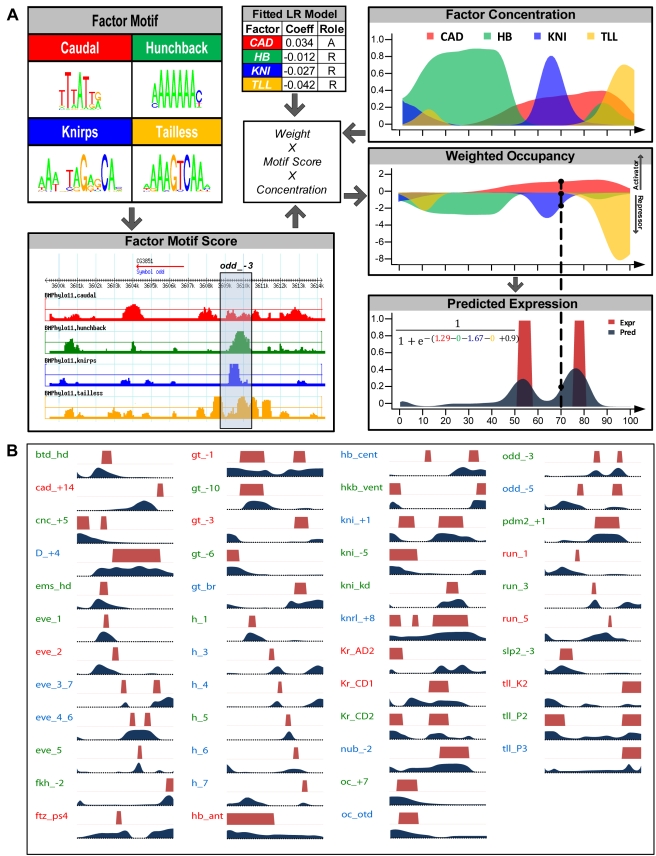
Logistic regression model and its performance on training data. (A) Components of the logistic regression model. For each transcription factor, its differential occupancy across the genome is described as a profile (“Factor Motif Score”) based on multi-species comparison of genomic regions using its DNA binding motif (“Factor Motif”). The contribution of the factor to the CRM's expression at that position (“Weighted Occupancy”) is described by the product of the factor's motif score in the given CRM (*odd_*−*3* in this example), its concentration (“Factor Concentration”) at a specific position along the A/P axis, and a weight assigned to the factor (“Factor Weights”). Contributions from all factors are added and transformed by a logistic function to predict the CRM's expression (“Predicted Expression,” dark blue). Factor weights are learned using a training set of CRMs with known A/P activity patterns. (B) Known (red) and predicted (dark blue) expression patterns, along the A/P axis, of 46 experimentally characterized CRMs. Heights of dark blue trace are proportional to the predicted expression level. Predictions deemed as being “good” (count = 20), “fair” (15), or “bad” (11) matches to known patterns (based on visual inspection) are indicated with green, blue, and red labels, respectively. In some cases, labels use abbreviated versions of CRM names.

We next used these binding site profiles to predict the potential transcriptional regulatory activity of any given genomic region. We reasoned that determining the potential of a region to generate patterned gene expression could help distinguish functional TF binding sites from regions that happened to have motif matches but were evolutionarily conserved for other reasons. A previous study [20] described a thermodynamic model that can recapitulate the expression activity of characterized CRMs. We developed a simpler, logistic regression model that could be readily adapted to multi-species analysis and genome-wide scanning and trained this model on a set of *Drosophila* CRMs. In any regression model, the parameters of the model are adjusted such that the output of the model (e.g., the predicted CRM activity at each A/P position in the embryo for the entire set of training CRMs) shows the greatest agreement with the training data (the experimentally determined expression profiles). Logistic regression models are a generalized version of linear regression where a sigmoidal (“logistic”) function is used to constrain the minimum and maximum output (e.g., CRM activity) to 0 and 1, respectively (see Figure S11). The logistic regression model used here combines weighted contributions from all TFs (using their expression and binding sites). The contribution of each TF is calculated heuristically as the product of its concentration and its binding affinity to the CRM (Figure 1A, middle panel and bottom right panel). The weight assigned to each TF indicates its regulating role—positive weights are used for activators and negative weights for repressors.

We used this model to predict the anterior-posterior (A/P) expression profiles of 46 experimentally characterized CRMs in the segmentation network [2], using multi-species motif profiles and expression patterns [2],[21] of the 10 TFs mentioned above. A binary representation of a CRM's activity profile along the A/P axis was modeled as a function of (i) each TF's motif score in the CRM's sequence and (ii) each TF's concentration value at that position (“bin”) along the axis, the bins being labeled from 1 (most anterior) to 100 (most posterior) (Figure 1A). The parameters of the model include a coefficient representing each TF's regulatory effect and a baseline expression value for each CRM (which is constant across all bins). These parameters were trained on the known expression profiles from the 46 CRMs. Visual inspection of the results (Figure 1B) indicates that the expression patterns predicted by the model are in good or fair agreement with the observed expression patterns for most of the 46 CRMs. By this qualitative assessment (which is consistent with the more quantitative assessment using “PGP scores” defined below), our method compares well with the results of the thermodynamic model, although a direct quantitative comparison is not feasible (Table S1). We tested for the possibility of the model “over-fitting” the data by comparing cross-validation results from the real data and randomized data and found a clear separation (*p* value = 1.2e-34) between the two (Figure S1), ruling out any significant over-fitting.

The above model provides “systems level” insights into the A/P network. We observed that coefficients for BCD, CAD, and FKH were fit to positive values while KNI, KR, GT, HB, TLL, HKB, and CIC were fit to negative values (Table S2A), broadly consistent with the activator/repressor roles known for these factors. (Although dual roles for some of these factors have been noted in the literature [22], our model learns a single dominant role consistent with the dataset.) We explored the effect of producing more complex relationships between TF expression and activity (by adding “second order covariates,” the squares of the term corresponding to each TF; see Methods) and found that a second order term for BCD improved the model (*p* value <E-16) by creating an anterior “dip” in the contribution of BCD to CRM activity (Figure S2). This broad anterior dip is not present in the BCD concentration gradient we used as input to the model. It may reflect previous observations that BCD levels appear higher than necessary for target gene activation by a simple BCD gradient model [23],[24]. Our model may not completely account for some aspect of down regulation of BCD target genes by the terminal patterning system, either by converting BCD into a repressor [25] or through regulation of other repressors [23],[24]. At the same time, the observation that second order covariates for the nine other TFs do not significantly improve the model's predictions suggests that the linear approximation provides a reasonable description of the CRMs' activities in terms of TF inputs.

We assessed the effect of using single or multi-species motif profiles in our CRM activity pattern prediction model and found that the multi-species Brownian Motion averaging-based profiles provided the best fit (Table 2). Improved performance with multi-species scores is broadly consistent with previous studies demonstrating that A/P CRMs with conserved activity patterns and similar binding site composition can be identified in related species [11],[26]. Interestingly, three individual modules, *eve_stripe4_6*, *gt_−1*, and *kni_+1*, have better predictions from the model trained with single species motif profiles (Figure S3). In at least one case, this discordance between the single species and multi-species predictions is mirrored in evolutionary changes within the CRM: there is experimental evidence that the *D. pseudoobscura* ortholog of the *gt_−1* module does not drive the posterior domain of *gt* expression that is observed for the *D. melanogaster* module (S. Sinha et al., manuscript in preparation). Thus, while the overall improvement in CRM activity predictions using multi-species profiles suggests that the majority of TF–CRM interactions in the A/P patterning network examined here are conserved, there are also examples of CRMs that have functionally diverged.

**Table 2:** Evaluation of different variants (column 1) of the logistic regression model, using three different goodness of fit measures: RMSE (Root Mean Square Error), average CC (correlation coefficient), and AIC (Akaike Information Criterion).

Model Implementation	RMSE	Avg. CC	AIC
Single species (without BCD^2^)	0.3135	0.43	3,028
Single species (with BCD^2^)	0.3088	0.46	2,962
Multi-species (simple averaging)	0.3090	0.47	2,966
Multi-species (BM averaging)	**0.3046**	**0.48**	**2,894**
ChIP-chip	0.3162	0.36	3,109

The best variant's score is shown in bold.

### Capicua Encodes the Torso Response Element Binding Factor

**Figure 2:**
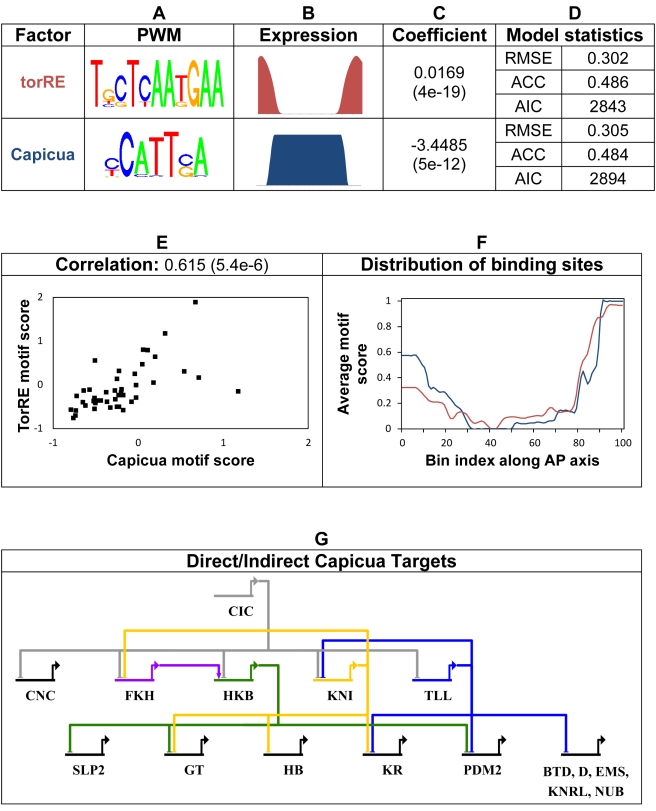
The role of transcription factor Capicua in A/P patterning. (A) The hypothetical activator TorRE and known repressor Capicua (CIC) have highly similar binding specificity (*p* value = 0.0012, Note 9 in Text S1), and (B) their expression profiles are perfectly complementary. (C) The regression model assigns highly significant weights to either motif and (D) the overall quality of fit is comparable between models that use one versus another. (E) Motif scores of TorRE and CIC are strongly correlated among the 46 A/P CRMs. (F) Average motif scores of TorRE (red) and CIC (blue) along the A/P axis (based on CRMs expressed at each position) are correlated, with high values at terminals. (G) Predicted regulatory network showing direct and indirect targets of CIC. Edges reflect a regulatory influence of CIC or its target TFs on any of the 35 CRMs included in the analysis, at an empirical *p* value threshold of 0.05. Directionality of influence is shown by arrow for activators (FKH) and flat line for repressors (CIC, HKB, KNI, TLL). Gray edges point to direct targets of CIC.

One of the strengths of the A/P network as a model system is that many relevant TFs have been identified in previous molecular and genetic studies. A potential unidentified factor was suggested by the characterization of a sequence motif “TorRE” (*Torso response element*) that is overrepresented in CRMs active at the anterior or posterior termini [27]. This motif and a hypothetical concentration profile (high at the two terminal regions) was previously used in a thermodynamic model of CRM function [20]. We considered the hypothesis that the TF Capicua (CIC) acts through the TorRE motif, suggested previously by [28] based on genetic data, and further examined in a later study [23]. CIC is a transcriptional repressor that is post-transcriptionally regulated in the embryo via degradation at the anterior and posterior termini in response to Torso signaling [28]. We determined the DNA binding specificity of CIC (Note 1 in Text S1) and found it to be similar to the TorRE (*p* value = 0.0012, Figure 2A), indicating that CIC can bind to most of the sites that contributed to identifying the TorRE. We found that the motif scores of TorRE and CIC are highly correlated across the 46 modules (correlation coefficient 0.62; *p* value = 5.4E-6; Figure 2E) and that CRMs with high motif scores (i.e., many potential binding sites) for either factor are mostly found at the terminal regions (Figure 2F). When the regression model is trained with either the TorRE motif or the CIC motif (and their respective concentration profiles, Figure 2B), the quality of fit is comparable (Figure 2C, 2D). Consistent with the complementary expression patterns for TorRE and CIC, CIC has a negative rather than positive coefficient, confirming that it generally acts as a transcriptional repressor. Adding TorRE to a model that already includes CIC leads to no significant improvement (unpublished data). These results indicate that CIC is the TorRE binding factor and that this factor acts by repressing target CRMs in the center of the embryo rather than activating targets at the termini. Individual direct and indirect targets of CIC are discussed below.

### CRM Discovery Based on Pattern Generating Potentials

**Figure 3:**
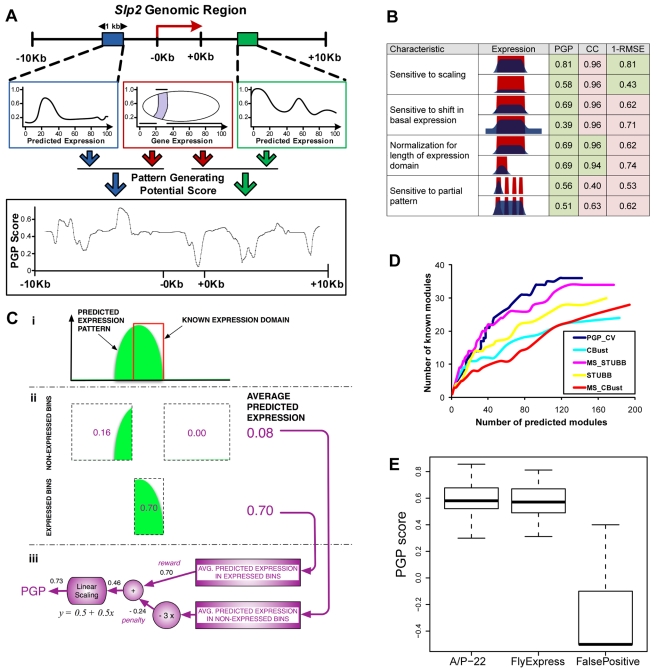
Pattern generating potential score. (A) Schematic for CRM discovery method. A genomic region (gene transcript, plus 10 Kbp upstream and 10 Kbp downstream) is scanned with a 1 Kbp window (filled rectangles). For each window, the predicted expression profile (open blue and green rectangles) is compared to the endogenous expression profile of the gene (open red rectangle, in center) to obtain the pattern generating potential (PGP) score, which is plotted (bottom panel) as a function of the genomic coordinate of the window. (B) Design features of the PGP score that distinguish it from the correlation coefficient (CC) or the root mean square error (RMSE). For each desired feature (“Characteristic”), two scenarios of comparison between known (red) and predicted (dark blue) expression profiles (“Expression”), along with PGP, CC, and 1-RMSE values, are shown. A perfect match would correspond to a value of 1 for each score. Cases where the value of a score in the two scenarios captures the desired feature are shaded in green. (C) Computation of the PGP score. (i) The predicted expression pattern (green) is shown along with the known domain of expression (red). (ii) The average predicted expression is calculated separately for domains of expression (the “reward” term) and of non-expression (the “penalty” term) and (iii) combined into the PGP score, by subtracting the penalty term from the reward term. The penalty term is assigned thrice as much weight as the reward term. The difference of reward and penalty thus computed is scaled linearly in the final step (“y = 0.5+0.5*x*”), giving us the PGP score. This scaling is merely a notational convenience (making the range of PGP scores fall between −1 and 1) and irrelevant to the way PGP scores are used in our pipeline. (D) Assessment of the PGP method and previous, binding site clustering-based methods for CRM prediction in the A/P-22 set. The number of known CRMs recovered (*y*-axis) in the top *k* predicted CRMs is shown, as a function of *k* (*x*-axis). The programs are: Cluster Buster (CBust) [9] and its multi-species version (MS_CBust, our implementation; see Note 8 in Text S1), STUBB [17] and its multi-species version (MS_STUBB, see Note 8 in Text S1), and PGP, evaluated within a leave-one-out cross-validation setting (PGP_CV). (E) PGP score distribution for CRMs predicted in the gene sets “A/P-22” (62 CRMs), “FlyExpress” (123 CRMs), as well as a “False Positive” set. The latter consists of eight experimentally tested sequences that contain a cluster of binding sites for A/P factors but do not drive any detectable expression in the embryo (Note 10 in Text S1). Medians, quartiles, and ranges are shown.

The ability to predict the spatial expression pattern driven by a module (CRM activity) suggests a method for discovery of novel CRMs: to scan the flanking genomic sequences of a gene for segments whose predicted activity pattern agrees with the gene's endogenous pattern. For this purpose, we developed a newly defined measure of similarity between expression profiles and its statistical significance; this measure is named the “Pattern Generating Potential” (PGP) (Figure 3A, Methods, Note 2 in Text S1). The scoring method was designed to: (1) be sensitive to both the shape and magnitude of the predicted expression profile, (2) avoid biases towards or against overly broad or overly narrow domains of expression, and (3) automatically allow sub-domains of a gene's expression pattern to be directed by the CRM (Figure 3B). To compute this score, we first calculate the average predicted CRM activity in domains of gene expression (the “reward” term) and domains of non-expression (the “penalty” term) and subtract the penalty from the reward, followed by a linear transformation generating PGP values between −1 and 1 (Figure 3C). An important feature of this score is that it can identify CRMs that contribute to only part of a gene's expression pattern (see below). When applied to the 46 CRMs used in the regression model above, the PGP score was highly correlated with our visual assessments of prediction success (Figure S4).

We tested this measure on the 22 genes (henceforth called “A/P-22”) regulated by the 46 CRMs described above. Expression data were obtained from whole embryo in situ hybridization images from BDGP (http://www.fruitfly.org/cgi-bin/ex/insitu.pl) and FlyExpress [29] (data available at http://veda.cs.uiuc.edu/lmcrm/). We scanned the control region of each gene (Note 3 in Text S1) with a sliding window of size 1 Kbp, predicted the A/P expression profile based on the motif scores in that window, and calculated the PGP (Figure 3A). An empirical *p* value representing the statistical significance of a putative module was estimated based on how frequently we observed a window with equivalent or greater PGP score in a genome-wide scan. Of the 62 modules predicted at a *p* value threshold of 0.015, 34 had significant overlap (>50%) with known modules, indicating 55% specificity at 74% sensitivity (Figure S5). Seventeen of the remaining 28 predicted modules overlapped the bound regions of at least one TF (ChIP data at 1% FDR from [6],[19]), indicating that the majority of predicted CRMs are functional and/or biochemical targets of A/P factors. Overall, we did not observe any systematic biases in the score, and modules with broad (“gap”) as well as sharp (“pair-rule” stripes) patterns were correctly predicted. The genomic location and predicted expression activity for each of these CRMs are available at http://veda.cs.uiuc.edu/lmcrm.

The 12 known modules not recovered included 10 that had either “bad” or “fair” predictions by the regression model (Figure 1), pointing out that CRMs whose expression is poorly predicted by the model are difficult to detect in the CRM search. For another CRM (*gt_*−*6*), the experimentally characterized activity pattern does not agree with the endogenous gene expression pattern we used (Note 4 in Text S1). In this case, the CRM activity pattern we used [2] may reflect either an experimental artifact or expression at a different embryonic stage. In only one case (*h_stripe1*), the PGP approach was unable to recover a module despite the training stage prediction being of high quality. Thus, most of the false negatives are likely to be due to the current limitations in the ability to predict CRM expression activity.

The results of this search were compared to two previously described CRM prediction programs, Cluster Buster [9] and Stubb [17], that search for clusters of binding sites for multiple TFs. To ensure that the performance of the PGP method was not influenced by including the same CRMs to train parameters that were then part of the predicted set, we used a cross-validation strategy where all known modules of a single gene were left out of the training phase before predicting CRMs within the control region of that gene. The PGP method performed better than both single and multi-species versions of Stubb and Cluster Buster (Figure 3D).

### A/P Patterning Is Frequently Regulated by Multiple, Functionally Similar CRMs

**Figure 4:**
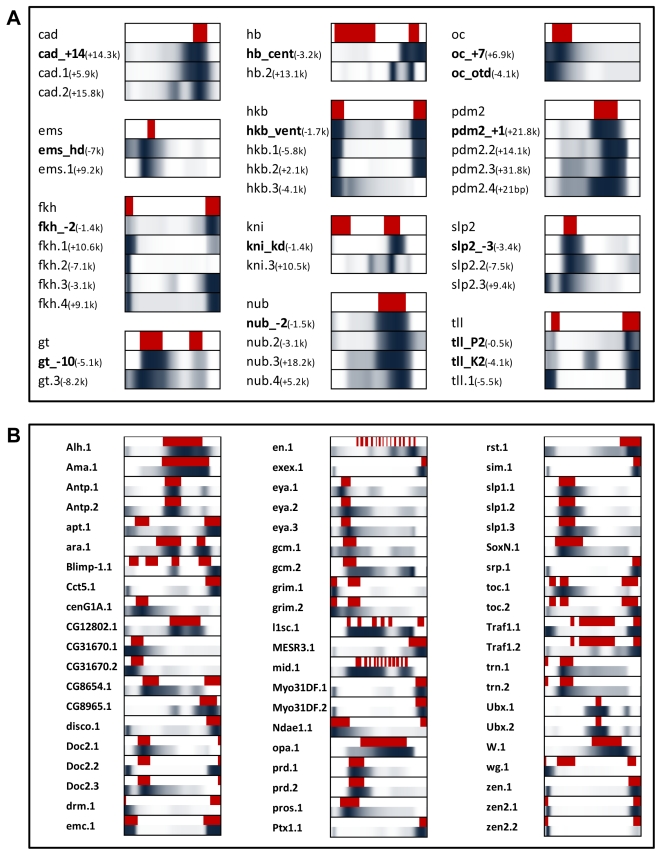
Expression patterns of predicted CRMs compared to known gene expression patterns. (A) Several genes in the A/P-22 set have two or more related CRMs (either predicted or known) that drive similar expression patterns. For each gene, the endogenous expression domain is shown (red), along with predicted expression profiles of CRMs (blue). Labels in bold indicate known CRMs. Predicted expression pattern is shown with color intensity proportional to expression value. (B) Gene expression pattern (red, top) is shown along with predicted expression pattern (dark blue, bottom) of 60 CRMs predicted in the FlyExpress set.

Unlike the other CRM prediction approaches, the PGP method predicts which aspect of the gene's pattern is regulated by an individual CRM, allowing the range of regulatory architectures for the A/P-22 genes to be examined: solitary CRMs, multiple CRMs contributing to distinct aspects of the pattern, or multiple “sibling” CRMs with a similar predicted activity. (We use the term “sibling” to indicate CRMs that may have effectively redundant activity within the context of our model, but possibly distinct contributions to the magnitude, temporal regulation, or robustness of patterned gene expression in vivo.) In our predictions, there was only one gene (*btd*) with a single predicted CRM; this prediction overlaps a known CRM (*btd_head*) driving the gene's expression. In all other cases, two or more modules were predicted in a single gene's control region. These included cases where distinct aspects of a gene's blastoderm expression pattern are captured by distinct predicted CRMs (e.g., five CRMs near the gene *eve*, including four known CRMs), a well-established phenomenon reported for primary pair-rule genes. We also found many cases of “sibling” CRMs, where multiple modules near a maternal/gap gene were predicted to drive highly similar expression patterns (Figure 4A). We considered whether possible false positive predictions could account for this observation; if the occurrence of a second, functionally similar CRM prediction in a gene's control region is an artifact of false positives, they should also be found near other randomly selected genes. However, we find that enrichment of functionally similar CRMs near the target gene is highly significant (*p* value = 4E-4, Table S3). Given the previous identification of “shadow” CRMs in the dorsal-ventral patterning network [30], the utilization of functionally similar CRMs may be a more common theme of *cis*-regulatory organization than currently recognized.

### Genome-Wide Discovery of A/P CRMs

We applied the PGP method to a larger collection of 144 genes with patterned expression along the anterior-posterior axis [12]. (A/P-22 genes were not included.) We automatically extracted the A/P expression profiles of these genes from the FlyExpress database [29], transformed the intensity values into binary expression domains (Methods), and identified flanking sequences with PGP at the same empirical *p* value threshold used above. (Predicted sequences that did not have above-average binding site presence for one of the activators in the model or for the broadly expressed activator Stat92E were discarded.) We identified 123 putative CRMs from 68 genes, henceforth called the “FlyExpress” gene set (data at http://veda.cs.uiuc.edu/lmcrm; the 60 most significant predictions are shown in Figure 4B). The distribution of PGP scores and their empirical *p* values is similar to that of A/P-22 and very different from that of bona fide non-modules that were identified as false positives in a cluster-based method to identify CRMs (Figure 3E and Figure S6). 44% of the predicted CRMs overlapped a ChIP-chip peak (at 1% FDR; 65% when considering peaks at 25% FDR; Table S4). The predictions included CRMs for genes with a single expression domain and genes with multiple expression domains (e.g., *slp1* and *ara*, respectively; Figure 4B). Among CRMs corresponding to genes with multi-domain patterns, 53% capture only one of the domains of the endogenous pattern (e.g., *drm*; Figure 4B) while 47% capture more than one domain (e.g., *emc*).

**Figure 5:**
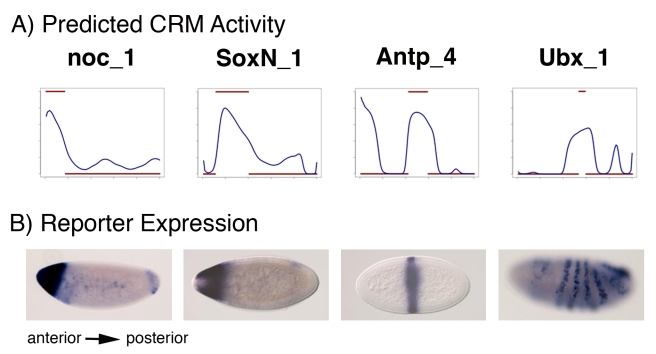
Experimental validation of predicted CRMs. (A) Predicted expression profiles are shown for genomic segments near four genes with A/P patterning from the “FlyExpress” set (*noc*, *SoxN*, *Antp*, and *Ubx*). The predicted expression is shown as a blue curve and the binarized blastoderm expression of the endogenous gene is shown as thick red lines. Additional reporters from three genes, *pdm2*, *emc*, and *apt*, were not active in early embryos (unpublished data). (B) The *cis*-regulatory activity of each region was tested in a transgene reporter construct. Spatial activity was determined by RNA in situ using a probe to a Gal4 reporter gene. Expression of the Ubx_1 reporter begins slightly after the blastoderm stage resembling the expression of the endogenous gene.

Sixteen of the above CRM predictions overlapped previously verified modules, of which 12 have blastoderm stage expression that agrees with the predicted expression profile from our model (Table S9). These provide an independent experimental validation for our CRM and activity prediction pipeline. In addition, we tested seven CRM predictions using new reporter transgenes. These lines were created as part of an ongoing project to systematically examine regulatory regions surrounding a subset of *Drosophila* genes with patterned expression in the nervous system [31]. Only predictions in intergenic or intronic regions of at least 10 Kbp were chosen for analysis. Selections included regions flanking genes manually annotated as “strong” or “weak” A/P patterned expression. 4 of 7 tested regions exhibited reporter gene expression patterns resembling the predicted pattern (Figure 5). For one of these, *Ubx*, the anterior boundary of reporter expression is in the correct region of the embryo, but initiation of the pattern is delayed relative to the endogenous gene and more strongly resembles the endogenous gene expression pattern at this slightly later stage; it has more posterior expression and a striped pattern likely reflecting the activity of later acting repressors not included in our model. Two of the remaining tested reporters (*pdm2* and *emc*) exhibit expression in the developing CNS, where many of the same TFs that regulate A/P patterning are re-expressed (unpublished data). It is possible that the same combinations of TFs that predict an A/P pattern in our model can act to direct patterned expression in the developing CNS. We note that the specificity we observed here (57%) is about the same as that recorded in our cross-validation tests on the A/P gene set.

We also examined the genome-wide locations of all segments with high PGP scores (and not just those located near the genes whose expression was modeled). We found these segments to be preferentially located near A/P patterned genes. However, we also observed a large number of segments (with high PGP) that are apparently not associated with patterned genes (Table S12). This suggests that the genome harbors a relatively large number of segments with PGP, and only a small subset of these actually realize this potential. This finding further supports our rationale of searching only in the neighborhood of a gene for segments with the potential to drive the gene's expression pattern.

### A Regulatory Network for Anterior-Posterior Axis Patterning in *Drosophila*

**Figure 6:**
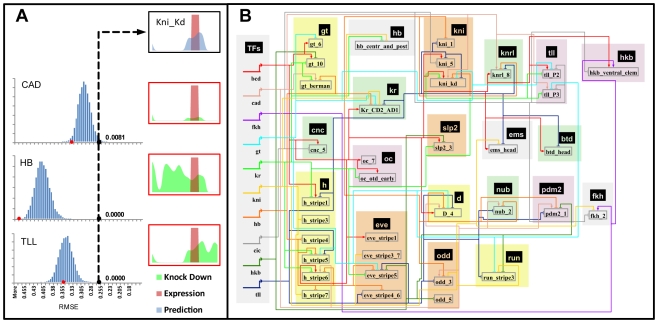
A gene regulatory network for A/P patterning. (A) Inference of TF–CRM interaction. For each motif, a histogram (blue) of RMSE scores (between real and predicted expression) is obtained from random permutations of the TF concentration profile, leading to a *p* value of the observed RMSE score (black dot on *x*-axis). Top right panel shows the true (red) and predicted (blue) expression profiles. Also shown is the effect of in silico “knock down” of each TF (panels on right, red border), and the corresponding RMSE score (red dot on *x*-axis of histograms). The expression profiles of the CAD activator and the HB and TLL repressors are shown in Figure 1A. (B) Predicted regulatory network for 10 TFs and 35 experimentally characterized CRMs. Edges reflect a regulatory influence from TF to CRM, at an empirical *p* value threshold of 0.05. Directionality of influence is shown by arrow for activators and flat line for repressors.

**Figure 7:**
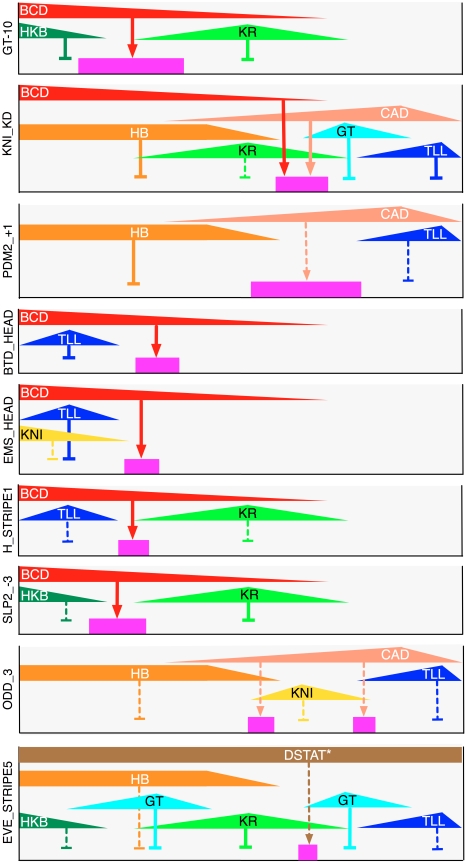
Examples of how maternal and gap patterned TFs together give rise to patterned expression. Shown are nine sample CRMs, their expression domains (in pink) along the A/P axis (left: anterior), their regulators (as per the predicted regulatory network of Figure 6B), and their respective expression domains (in color code matching that of Figure 6B). Arrows indicate activation and barred lines indicate repressive influence. Repressor domains shown are required to be overlapping with an activator's domain of influence. Solid edges indicate that the regulatory influence is supported by previous experimental evidence in the literature, while dashed edges indicate novel interactions. Labels of TF expression domains are in black or white for better color contrast and have no semantic difference. *The edge between DSTAT and *eve_stripe5* is not based on our model predictions (since DSTAT is broadly expressed and was not included in the model) but on the presence of DSTAT binding sites (motif score greater than 4 standard deviations above genomic mean) in the CRM.

Unlike binding site clustering methods, the PGP method uses both the binding specificities of TFs and their expression pattern to predict the activity pattern of a CRM. Using the PGP method, it is possible to computationally assess the contribution of each TF to the CRM by asking if altering the expression of the TF affects the quality of the prediction. We used this strategy to infer direct regulatory interactions between TFs and CRMs, depicted as edges in the transcriptional regulatory network. To visualize the effect of removing an individual TF from the model, we simulated a “knock down” of the TF (by setting its expression to 0) and compared the predicted CRM expression in this “in silico mutant” background and in “wild type” (Figure 6A, knock down patterns shown in green). Unlike traditional in vivo genetic assays, where observed changes may be the indirect effect of mis-regulation of other genes, this approach examines the direct contribution of a TF to a specific CRM. In order to assign a statistical significance to this contribution, we developed an alternative procedure (Methods and Figure 6A): CRM activity predictions were generated using random permutations of the TF's concentration profile and compared to the “true” activity, thus creating a null distribution of similarity scores (depicted in blue). The score obtained with the actual profile (black dot) was compared to this distribution, generating an empirical *p* value. When there are few binding sites in the CRM, the TF pattern has little influence on CRM predictions and the null distribution of scores is very narrow (unpublished data). When there are more binding sites in the CRM, there is a broader distribution of similarity scores from the random profiles, and the position of the actual profile within this distribution reflects the combined contribution of the binding sites and the normal TF expression pattern on CRM activity. Using this procedure to infer a *p* value for every TF–CRM combination, we constructed a transcriptional regulatory network (Figure 6B, Figure 7) involving the 35 CRMs where the model's quality of fit had been “good” or “fair” (Figure 1B).

A total of 102 regulatory edges were predicted (at *p* value <0.05) between the 10 TFs and 35 CRMs, revealing a very dense network. (See http://veda.cs.uiuc.edu/lmcrm.) 82 edges were supported by ChIP-based evidence of occupancy at the strongest level (1% FDR). 63 of the 102 edges have been previously reported in the literature, mostly by examination of CRM activity in mutant embryos lacking the TF (Table S5). In some cases, confidence in experimentally determined TF–CRM edges is further increased by in vitro confirmation of TF binding sites by DNaseI footprinting. For 12 of the 35 CRMs analyzed above, the FlyReg database [32] catalogs at least one such interaction with either BCD, CAD, KR, KNI, HB, GT, or TLL. These validated TF–CRM edges were significantly enriched in our network (Hypergeometric test, *p* value = 0.0026) (Figure S7).

This network model can address specific questions about the role of individual TFs in A/P patterning. For example, the concentration of the repressor CIC is a direct output of the terminal patterning system [33], but it is not known whether this mechanism acts solely by determining the terminal expression patterns of TLL and HKB. Terminal gene expression could be either entirely regulated by these factors or the terminal system might also directly regulate additional targets via CIC. The regulatory network model predicts that CIC directly targets at least six CRMs corresponding to five distinct genes—*tll* and *hkb* as well as *cnc*, *fkh*, and *kni* (Figure 2G). Thus, this result extends our observation above that CIC binds to the Torso response element and indicates that control of CIC levels by the terminal regulatory system indirectly regulates many genes via *tll* and *hkb* but also has some additional direct targets besides *tll* and *hkb*. Existing experimental evidence also points to a role of the terminal system in regulating these CRMs or their associated genes [34]–[36], although evidence of direct influence has been missing. This finding complements our current understanding of the terminal patterning system, which has thus far been shown to act only through the TFs TLL and HKB [36],[37].

We used the above statistical procedure to construct a regulatory network for all of our CRM predictions (62 in the A/P-22 set, 123 in the FlyExpress set); the TF–CRM interactions composing this network are cataloged in Table S6. Analysis of the predicted network reveals several common patterns. A recurring theme in the TF–CRM interactions is potential “auto-regulation” by activators. For example, all three predicted modules near the *cad* gene had significant regulatory input from CAD, and in each case, this predicted auto-regulation was supported by ChIP data (at 1% FDR). Similarly, four out of five predicted modules for *fkh* are predicted to have FKH-driven activation. *fkh* auto-regulation (in salivary glands) has been experimentally shown [38]. On the other hand, auto-regulation by repressors is not seen in our predictions, as anticipated. Another common theme observed was that of mutual repression by pairs of TFs, e.g., HB–KNI, GT–KR, KR–KNI, HB–KR, GT–KNI, and TLL–KR, some but not all of which were reported previously [22],[39]–[41]. We also used edges of this network to characterize the “complexity” of CRMs. About three TFs on average had edges leading into each CRM, for most target patterns, except that CRMs driving expression at the anterior seem to have relatively low complexity and those active in bins 80–90 have slightly greater complexity (Figure S8). When we examine the degree distribution of the network from the perspective of the TFs, all 10 TFs contributed almost uniformly to the predicted CRMs (Figure S9).

We compared the above-mentioned network to that predicted by Stark et al. [13], which was based on the presence of conserved (predicted) binding sites in 2 Kbp promoters of all *Drosophila* genes. However, we found very little overlap between the two networks (Note 5 in Text S1), which we attribute to the fact that only a small percentage of our predicted CRMs (and of experimentally validated A/P CRMs) are located in the 2 Kbp regions immediately upstream of genes.

### Comparable Performance of Binding Site Profiles and ChIP Data within Models of Transcriptional Networks

Genome-wide ChIP assays provide the location and strength of TF occupancy in vivo. Compared with cell culture or yeast experiments, intact organisms represent potentially more challenging contexts to interpret ChIP data since TF expression can vary across space and time. A recent analysis of CRMs acting in mesoderm development demonstrates that time course ChIP data can predict multiple distinct classes of CRM activity patterns in whole embryos [8]. In contrast, complementary computational methods lack the in vivo context of ChIP but can provide a potentially general approach to predict regulatory networks, even in cells and tissues that are difficult to characterize experimentally. A high-quality genome-wide ChIP dataset is available for eight of the A/P TFs during early embryogenesis [6],[19]. In the previous sections, we have used this dataset to confirm that a majority of the PGP-derived CRM predictions correspond to in vivo occupancy by one or more TFs. In this section, we evaluate if the TF–CRM interactions predicted by the PGP method can approach the quality of predictions derived from ChIP data.

#### Predicting expression patterns

For the eight TFs for which ChIP data are available [6],[19], we replaced the motif score profiles with ChIP scores and retrained the regression model using these data. By statistical measures, the overall quality of fit of the ChIP-based model was inferior to that with multi-species motif profiles (Table 2, e.g., average correlation coefficient of 0.36 compared to 0.48 achieved with motif profiles). Specific examples reflecting an inferior model include assigning a positive coefficient (activating role) to the well-known repressor GT (Table S7) and less statistically significant coefficients for other repressors (KR, KNI). Thus, in the context of this experimental system, computational binding site prediction together with comparative genomics may have equal or even greater utility than the use of ChIP-based measurements of TF occupancy.

#### Predicting functional occupancy

Schroeder et al. [2] presented a method for a systems-level perspective on the occurrence of functional binding sites in segmentation modules. Their method sums the motif scores for a TF for all of the CRMs active at each position along the A/P axis. They noted that for CRMs that drive expression in the same domains as a TF, there is the expected overrepresentation of binding sites for activators (positive correlation) and an under-representation of binding sites for repressors (anti-correlation). These correlations should be more pronounced if functional TF binding in the CRMs is more accurately described. We examined these correlations with our multi-species motif profiles as well as with ChIP-chip scores of occupancy (Figure S10). For six of the eight factors, the degree of correlation was comparable for either approach; however, for KNI and GT, the motif profiles showed a marked improvement in anti-correlation with the respective concentration profiles. This analysis further supports the conclusion that motif scores based on cross-species comparison may better estimate the regulatory effect of a factor than ChIP data that measure biochemical occupancy but lack the evolutionary filter for regulatory function.

#### Predicting regulatory TF–CRM interactions

Of the 102 significant regulatory network edges predicted above (Figure 6B), 94 correspond to the TFs BCD, CAD, HB, GT, KR, KNI, HKB, and TLL for which ChIP data are available. 80 of these 94 predicted edges have ChIP-based support at the highest level of confidence (1% FDR), seven more at the 25% FDR level, and only seven are not corroborated by ChIP data. However, such extensive agreement between motif-based edge predictions and ChIP data could reflect widespread biochemical occupancy [6] that does not have a regulatory function in this context. Indeed, there is a strong tendency for occupancy by most of the eight factors in the set of known modules: of all possible TF–CRM combinations, 76% had ChIP support at 1% FDR.

**Figure 8:**
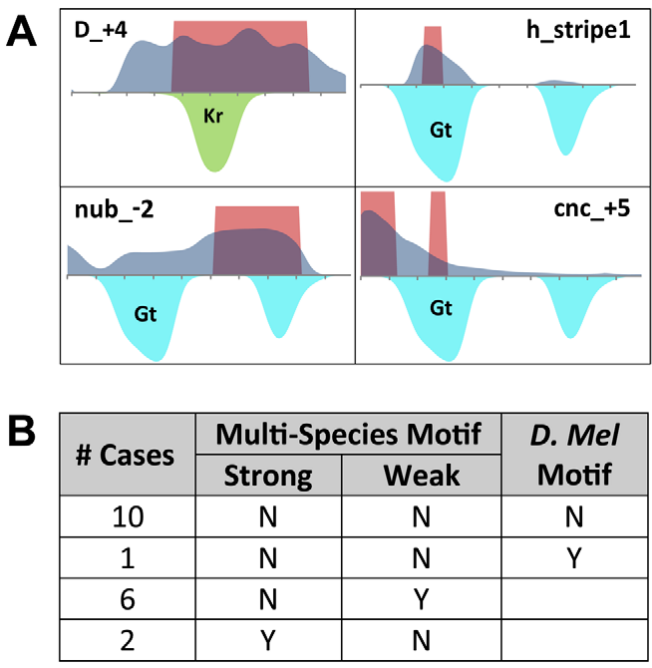
Incongruous occupancy by repressors. (A) Four examples of incongruous occupancy, from ChIP data, by repressors (GT or KR) in known CRMs. Shaded areas above horizontal axis indicate domains of expression driven by the CRM (red = real, dark blue = predicted). Shaded areas below axis are regions where a repressor (GT in blue or KR in green) is present and will thus inhibit expression if it occupies the CRM. (B) Motif presence or absence in cases of incongruous occupancy indicated by ChIP. “Multi-species Motif” shows whether the multi-species motif score is strong (>2 standard deviations above genomic mean), weak (above genomic mean), or neither. “D.mel Motif” shows, for cases where the multi-species score indicates motif absence, whether motif score in *D. melanogaster* is above genomic mean or not.

We compared predicted TF–CRM edges based on ChIP-chip data to the FlyReg literature-based regulatory network mentioned above, using either the 1% FDR or top 100 ChIP scores as a cutoff for interactions (Figure S7). Again, the trend of frequent TF occupancy in ChIP data had a significant impact on this analysis. The PGP-based regulatory network actually exhibited a greater enrichment for the validated TF–CRM interactions than the ChIP-derived network, primarily by predicting fewer interactions but with higher precision. Among the examples of interactions predicted by ChIP, but not PGP, we found multiple surprising examples of ChIP data indicating TF occupancy that should adversely affect the module's expression profile. Specifically, we identified CRMs with ChIP signals for the repressors KR, KNI, or GT, and whose activity domain overlaps the bound repressor. Overall, we found 19 such cases of apparently “incongruous” occupancy (Figure 8, Table S8). In 17 of these cases, we did not find corresponding support for evolutionarily conserved binding sites from our multi-species motif profiles. These examples indicate a discrepancy between motif-based evidence and ChIP evidence and appear to be cases where the observed biochemical occupancy does not act to shape the activity pattern of the CRM.

## Discussion

As large numbers of genome sequences become available, annotation of how different genomic segments contribute to organismal function remains a central challenge. Despite the relative simplicity of the genetic code, annotation of the protein-coding regions of large genomes has undergone continued revision as new experimental datasets and computational approaches have been developed. Computational annotation of CRMs is significantly less advanced, in part due to the incomplete description and complexity of metazoan TF-DNA binding specificities. However, even after determining binding motifs for the central regulators of *Drosophila* anterior-posterior patterning network [14], we found it difficult to use existing clustering strategies to systematically search for the targets of these factors. Here, we describe an alternative strategy—use binding site motifs to predict the A/P activity pattern for a given DNA sequence and determine the similarity of the predicted activity pattern to an experimentally determined expression pattern. The PGP can be used to annotate the non-coding genome, similar to the “regulatory potential” score of [42]; unlike the regulatory potential, which generally classifies non-coding sequences as regulatory or neutral, PGP ranks sequences by their ability to contribute to the specific expression pattern of a nearby gene. It further facilitates a quantitative inference of TF–CRM interactions, whose validity may then be assessed through in vivo observations. We have specifically applied this approach to the A/P network, but it should be applicable to any system in which adequate expression data are available for relevant TFs, CRMs, and target genes.

One key distinction between using PGP to characterize CRMs instead of binding site clusters is that this method can automatically select appropriate combinations of TFs to contribute to a CRM's activity. By only considering TFs expressed at the appropriate time and place, this approach partly addresses issues associated with TF specificity overlap and pleiotropy. A second advantage is the rich class of expression patterns with which it may be used. The current implementation accommodates expression states composed of any combination of 100 positions along the A/P axis and can be expanded in a straightforward way to include additional spatial and temporal dimensions. These expression patterns can even be the result of automated image-processing pipelines, such as the one used here for A/P patterns. This is in contrast to the more limited classes of manually determined expression patterns considered in previous studies [8]. The regression model also has the advantage that the explicit activity pattern predictions are easily interpreted, compared to other machine-learning techniques such as Bayesian networks [43] or support vector machines [8]. As noted above, replacing binding site profiles with ChIP-based occupancy data in our model did not lead to superior predictions. This negative result is somewhat contrary to the findings of Zinzen et al. [8], who exploited ChIP data on five TFs (at five different time points) to successfully predict spatio-temporal expression patterns of many CRMs involved in mesoderm specification. Integration of both ChIP and motif presence information may hold the key to significantly improved predictions and will be an exciting area for future research.

Generating the experimental datasets required to apply this method more broadly should be feasible with current technologies. The bacterial one-hybrid system and other methods should generate DNA binding specificities for most *Drosophila* TFs [14]–[16],[44]. Systematic determination of the temporal and spatial expression patterns of TFs is critical and not a minor task; however, it should be far more straightforward than applying genome-wide ChIP methods to the many different possible cell types present at different developmental stages. In addition to TF binding specificities and expression patterns, two other datasets are required. First, large-scale descriptions of gene expression patterns must be available; these are currently being generated for the *Drosophila* embryo [29],[45],[46]. Second, a training set of CRMs with diverse activity patterns must be identified; for the *Drosophila* embryo, these can be curated from the literature [47] or generated in moderate to high throughput reporter studies [31]. While we have treated CRM and gene expression patterns as binary values at a single developmental time point, quantitative spatial and temporal expression data are readily accommodated in this approach and should capture more comprehensive and subtle aspects of transcriptional regulatory networks. We note that the specific components of the model used here for predicting segmentation modules may change as more genomes and relevant TF motifs are characterized in the future. At the same time, our tests suggest (Tables S10, S11) that including more genomes (and to some extent additional motifs) may not lead to dramatic improvements.

The logistic regression model used here is very similar to the more popular linear regression model [48], combining weighted contributions from all TFs, except that the logistic model imposes the combined output to saturate at high values (Figure S11). This model is “simpler” than a previously published thermodynamic model to predict regulatory function from sequence [20], in that it has fewer parameters to be trained from data. At the same time, it performs well compared to the thermodynamic model and has the added advantages of easily incorporating multiple species comparisons and of computation that is orders of magnitude faster. This enables fast, genome-wide prediction of other CRMs, examination of the effect of each motif on each putative CRM, and empirical assessment of its statistical significance through permutation tests. However, the regression model does not incorporate known mechanistic features of CRM function, such as cooperative TF binding. More detailed models of CRM function have been developed for individual enhancers [40],[49],[50], which can accurately describe changes in CRM activity over developmental time or due to mutation. In principle, these models or other approaches to capture how binding site arrangements contribute to expression could replace the regression model in the overall framework to measure PGP. Models with additional parameters may provide better predictions but require additional prior knowledge, while models with fewer parameters may generalize better. We also note that the motif scores used in our model are based on evolutionary conservation at the ∼500 bp resolution and are thus robust to local turnover of sites [51]. The approach is also applicable with single-species motif scores (although this led to poorer performance in our setting), which may be significant for discovery of CRMs that are not as well conserved [52].

This analysis of the *Drosophila* A/P patterning using PGP is the most complete description of this network to date. The quantitative descriptions of how TF inputs generate the activity pattern of specific CRMs and the explicit predictions of individual TF–CRM interactions provide a level of detail not typically generated by other approaches. In this study, we have highlighted a few specific novel observations on the predicted regulatory network, such as which genes regulated by the terminal system are direct or indirect targets of CIC. We have also identified and experimentally confirmed the activity of four new CRMs of the A/P patterning network, regulating the genes *Antp*, *noc*, *SoxN*, and *Ubx*. In addition, we identified several general conclusions about the network, including the frequent occurrence of positive autoregulation by activators and mutual repression by spatially adjacent repressors. One of the most striking results is how often individual genes have multiple CRMs predicted to direct the same embryonic expression pattern. Individual examples of such “sibling” CRMs have been previously described in both the A/P and D/V embryonic patterning networks, but the current analysis indicates that they may be more frequent than previously appreciated. (A more complete experimental analysis of this aspect of *cis*-regulatory architecture will be required given the observation that at least some of these CRMs are in fact “cousins” that appear to use similar TF binding sites to drive patterned expression in a different tissue.) Application of the PGP method to other transcriptional regulatory networks should reveal if similar overall regulatory themes act in other developmental contexts.

Recent ChIP-chip analysis of multiple TFs regulating *Drosophila* embryonic patterning provides a quantitative dataset to compare with our computational approach [6],[19]. Overall, ChIP data suggest far more binding events than expected to be required to directly control patterned gene expression [6], consistent with earlier predictions of widespread genome binding by TFs [53],[54]. Presumably, as long as occupancy does not interfere with patterning, it can be tolerated. In contrast, computational TF binding site profiles incorporate multiple species comparisons to probe where TF binding sites are under evolutionary selection, which should reflect a conserved role in patterning. In our comparison of ChIP data and TF binding site profiles for the A/P network, we find that evolutionarily conserved binding sites provide greater specificity and that this leads to better gene expression prediction models and a greater enrichment of known TF-CRM interactions. Interestingly, we found several examples of disagreement between motif-based and ChIP-based prediction of binding where the ChIP occupancy appears to be antagonistic to the known activity pattern of the CRM. For future work in cells where high-quality ChIP data are available, integrating ChIP scores and multi-species motif profiles may allow higher confidence predictions of CRM position, function, and regulation by combining both experimental evidence for availability and occupancy with evolutionary evidence for function [13],[55]. For cells where ChIP data are not available, determining the PGP of genomic regions can provide a general strategy to annotate regulatory regions.

In summary, this work presents a general computational framework for analyzing transcriptional regulatory networks through a systematic integration of sequence (from multiple species), expression, and TF binding specificity data, all of which are hallmarks of the genomics toolkit available today. Application of the framework provides systems-level insights into the regulation of anterior-posterior patterning in the *Drosophila* embryo.

## Methods

### Motif Score Profiles

All motif profiles are made available through the Genome Surveyor interface at http://veda.cs.uiuc.edu/lmcrm.

### Brownian Motion Averaging

Given a phylogeny and a profile value for each leaf node or extant species, our task is to compute an evolutionary average of the given values. Following [56], we consider a random variable evolving according to a Brownian Motion process along each branch, with the process on each branch being conditional on the value of the variable at the parent node of that branch. The temporal expectation of this random variable, over all branches, is the desired average. Exploiting the observation that the random variable has a Gaussian distribution at every (non-root) node with mean and variance defined by the value at the parent of that node, researchers have shown [18] how this temporal average may be calculated in time O(n^2^), where n is the number of species. We developed an alternative, O(n) algorithm for this purpose based on the upward-downward algorithm paradigm for trees (Text S2) [57].

### Evaluation of Motif Score Profiles Using ChIP-Chip Data

The top 100 bound regions of a TF, each defined as the 500 bp centered on a ChIP peak, were used, along with 5,000 length-matched sequences selected at random from non-coding regions. For each sequence, the motif score was computed, and the sets of scores for the bound and random regions were compared using the Wilcoxon Rank-Sum test.

### Logistic Regression Model

The basic model for predicting CRM expression patterns is as follows:
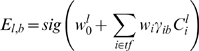
(1)where 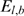 is the expression value (between 0 and 1) of the CRM *l* in bin *b* (the A/P axis is divided into 100 equal bins), 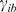 is the concentration of factor *i* in bin *b*, 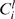 is the motif score of factor *i* in the CRM *l*, 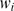 is the regression coefficient for factor *i*, 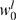 is the “basal” expression level of CRM *l*, *sig*(x) is a “sigmoid” function 1/(1+exp(−x)).

The free parameters are 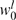 (one for each CRM) and 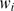 (one for each factor). Use of the CRM-specific parameter 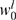 is motivated by (i) the fact that the discrete (0/1) expression values that form the desired output do not reflect the variation in basal gene expression levels and (ii) an opportunity to compensate for, at least partially, the lack of complete knowledge of relevant TFs, especially of ubiquitous activators and/or repressors. Note that the concentration and motif score terms occur together (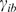 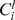) and this product is called the “covariate” of factor *i* for CRM *l* in bin *b*. An additional higher order term, called “BCD^2^,” is used in our model. BCD^2^ is the square of the covariate “BCD” for the factor BCD. Utilizing the glm (generalized linear model) function in R's “stats” package [58], we trained the parameters of the model using iteratively reweighted least squares (IWLS) to minimize the error between predicted and true expression values. The ratio between the trained parameter and its estimated standard error was treated as a *z* score for calculating its statistical significance. The overall quality of fit of the model to the data was measured by standard statistics such as the root mean squared error (RMSE), mean Correlation Coefficient (CC), and the Akaike Information Content (AIC) (Note 6 in Text S1). All analyses were performed within the R programming environment.

### PGP

Given a predicted expression profile (real numbers between 0 and 1 for each bin along A/P axis) and an endogenous expression profile (0 or 1 values for each bin), we defined the PGP score as follows:
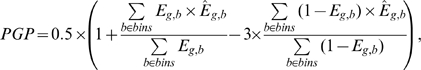
(2)where *E_g,b_* is the expression value (0 or 1) of the gene *g* in bin *b* and 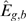 is the predicted expression value (between 0 and 1). This score ranges from −1 to +1. It rewards correctly predicted domains of expression and penalizes false prediction of expression. If the endogenous profile has multiple domains of expression, a subset of those domains are selected based on the predicted profile and then compared to the predicted profile using PGP. See Figure 3B, Figure 3C, and Note 2 in Text S1 for additional details.

### Regulatory Network Edge Prediction

First, we computed the “root mean square error” (RMSE_wt_) between the model's prediction in “wild type” and the true expression. Next, the concentration profile of a factor was permuted randomly and the RMSE was determined for each permutation. Repeating this step 1,000 times, we obtained an empirical *p* value of RMSE_wt_, which represents how important this factor (in particular, its concentration profile) is to the CRM's expression pattern.

### Fly Express Data Mining

A/P axis expression profiles were calculated from ∼36,800 BDGP in situ expression images (lateral orientation). The images were first converted into a standardized format and aligned using the FlyExpress image processing pipeline [59]. The resultant 128×320 standardized images were then manually inspected and corrected, as necessary. For generating an A/P expression profile, the expression values were calculated for each of the 320 points along the A/P axis by taking the average of the intensity values within a window around the middle of the Dorsal-Ventral axis. All images for a given gene and developmental stage 4–6 were visually examined to identify those with A/P patterns and select a representative image, whose A/P profile was further discretized into domains of expression and non-expression along 100 equal-sized bins along the axis, as described in Note 7 in Text S1.

### In Vivo Analysis of CRM Activity

Genomic regions encompassing predicted CRMs were tested as transgene reporter constructs as described previously [31] using labeled RNA probes to assay expression of the Gal4 reporter gene by in situ hybridization to mixed stage embryos. Candidate CRMs for the following genes were tested (with release 5 coordinates for the tested fragment): *noc* (2L:14487040–14490562), *SoxN* (2L:8831242–8833223), *Antp* (3R:2774228–2775839), *Ubx* (3R:12503151–12505092), *apt*, (2R:19455701–19458627), *emc* (3L:745029–746591), and *pdm2* (2L:12669616–12672809).

## Supporting Information

Figure S1 **Distribution of prediction error on test data obtained from 1,000 replicates of 2-fold cross-validation, on the original data (red) and randomized data (blue).** The cross-validation was done by designating a randomly chosen half of the 4,600 points (46 CRMs×100 bins) as training data and testing the trained model's predictions on the remaining half. Prediction error was defined as the root mean squared error on the test points. A randomized dataset was constructed by randomly permuting the matching between CRMs and their expression profiles. The model is never able to achieve, for the randomized data, the kind of low error rates it achieves on real data (*p* value = 1.2e-34 based on one-tail Wilcoxon rank-sum). This strongly suggests that the regression model is not “over-fit” to the data of 46 CRMs and their respective expression patterns.
(0.17 MB TIF)

Figure S2 **Concentration profile of BCD in our model (with anterior on left).** Shown is the effect of BCD, blue, the effect of quadratic form of BCD, green, and the combined effect of both terms, brown, for the CRM *btd_head*.
(0.11 MB TIF)

Figure S3 **Comparison between predictions with multi-species (BM) and single species (** ***D. mel*** **) motif profiles for three modules in which single species perform better than multi-species.** The white, yellow, and orange colors represent non-significant, moderate (above genomic mean), and significant (>2 standard deviation above genomic mean) motif counts, respectively.
(0.20 MB TIF)

Figure S4 **The PGP score, when applied to measure the similarity between predicted and known expression patterns of a CRM, is highly correlated with our visual categorization of the predicted expression as being a “good,” “fair,” or “bad” match to the known expression pattern.** These visual categorizations were catalogued in Figure 1B. Shown here is the distribution of PGP scores (means and 25th and 75th quartiles) for each of these three categories, which have 20, 15, and 11 CRMs, respectively.
(0.10 MB TIF)

Figure S5 **Assessment of the PGP method on A/P-22 set using model trained on all known CRMs.** Graph shows the number of retrieved known CRMs (*y*-axis) as a function of the number of predicted CRMs (*x*-axis). The dashed line indicates an empirical *p* value threshold of 0.015, which was used in our final predictions.
(0.11 MB TIF)

Figure S6 **PGP empirical** ***p*** **value for CRMs predicted in the gene sets “A/P-22” (62 CRMs), “FlyExpress” (123 CRMs), as well as the “False Positive” set of eight CRMs.** The latter consists of eight experimentally validated CRMs that do not drive any detectable expression in the embryo. These are *nub_+5*, *pdm2_+3*, *pdm2_+5*, and *pdm2_+8* from [2] and PCE8008, PCE8021, PCE8023, and PCE8007 from [11]. Additional bona fide CRMs from [11] are not considered because their neighboring genes are not A/P patterned, which implies that those non-CRMs will not even receive a score under our PGP scheme.
(0.11 MB TIF)

Figure S7 **(Top) Overlap between the predicted and experimentally validated regulatory network edges.** FR represents 33 experimentally validated edges (from FlyReg) between 12 known CRMs and 7 TFs (BCD, CAD, KR, KNI, HB, GT, and TLL). LR is the set of regulatory edges predicted by our linear regression model for the same set of CRMs and factors. “ChIP” refers to edges inferred based on TF occupancy revealed by ChIP-chip (at 1% FDR). “ChIP100” is the same dataset, except that only the top 100 bound regions are considered. The total number of possible edges is 12×7 = 84. (Bottom) Sensitivity, precision, and Hypergeometric *p* values of overlap of each method's edge predictions with the “test” regulatory network comprising 33 experimentally validated edges from FlyReg.
(0.15 MB TIF)

Figure S8 **(Top) Distribution of the average number of motifs present (motif score >0) in all predicted CRMs driving expression in a bin along the A/P axis.** (Bottom) Average number of motifs (TFs) with “regulatory edges” to all predicted CRMs driving expression in a particular bin along the A/P axis.
(0.17 MB TIF)

Figure S9 **Shown is the fraction of genes/CRMs targeted by each transcription factor (as per our regulatory edge prediction method) in the A/P-22 and FlyExpress gene sets.** The number of interactions with genes/CRMs is almost uniform across TFs.
(0.15 MB TIF)

Figure S10 **The contribution of individual transcription factors to the 46 known CRMs, as a function of the position along the A/P axis where the CRMs drive expression.** For each position (*x*-axis), shown are the average motif score (black) and ChIP-chip score (blue) of the factor in CRMs driving expression at that position. The red curve is the concentration profile of the transcription factor. Note that for KNI and GT, the black curve (motif score based) shows much better anti-correlation with the TF profile than does the blue curve (ChIP based).
(0.32 MB TIF)

Figure S11 **The logistic function used in our logistic regression model.** The logistic function is a commonly used S-shaped function that takes values between 0 and 1. The logistic function used here is y = 1/(1+exp(−x)).
(0.07 MB TIF)

Table S1 **The first row summarizes the visual classification of the quality of fit (as “good,” “fair,” or “bad”) between the model's prediction and known expression patterns of 46 known CRMs.** The second row shows the results of a similar visual classification of predictions from a previous thermodynamic model of [20]. These results should not be interpreted as a strict comparison of models, since the numbers and identities of CRMs used in the two analyses are slightly different, the numbers of motifs used as input are different, and the numbers of free parameters trained by the models are widely different. Moreover, the predictions from the Segal et al. model are not publicly available in a machine-readable format.
(0.03 MB DOC)

Table S2 **(A) Covariates of our model and the significance of their contributions: the first column lists the model covariates (predictors).** The second column is the trained value of the regression coefficient (wi) of each covariate, and the third column is the significance of the coefficients (computed as described in Methods). Grayed rows correspond to TFs inferred to be activators (positive coefficients), while others are repressors (negative coefficients). (B) The trained value of the “baseline” parameter for each CRM: the baseline values range from ∼−4.5 to ∼3 with an average of −1.4
(0.06 MB DOC)

Table S3 **Statistical evidence for prevalence of functionally redundant (“sibling”) CRMs near maternal and gap genes.** CRMs driving a particular aspect of a target gene G's expression pattern were predicted genome-wide using the PGP method. These CRMs may be located in the control region of gene G itself, or not. Those located in the control region of the target gene itself are called “real” and the rest are called “random.” The one or more predicted CRMs in the control region of the same gene (which may or may not be the target gene), driving the same expression pattern, are defined as a CRM set. A CRM set may be “solitary” (cardinality of one) or “redundant” (cardinality of more than one). The predicted CRMs constituting a redundant CRM set are functionally redundant CRMs, potentially. Also, as noted above, a CRM set may be “real” (if located in the control region of the target gene) or “random.” A 2×2 contingency table is thus defined and its statistical significance estimated by the Fisher's exact test. The *p* value obtained for this table is 4.0E-4, strongly suggesting that “real” CRMs (i.e., predicted CRMs more likely to be true positives) are enriched for the property of having functionally related partners (“siblings”).
(0.03 MB DOC)

Table S4 **Properties of FlyExpress predicted modules, shown for various selection criteria.** The grayed row corresponds to the criteria used in the paper. The first column shows the PGP *p* value threshold used in prediction of modules. The second column lists the type of additional filter applied for selecting modules: “Best of each gene” indicates that only the module with the lowest *p* value for the gene was selected. “Activator presence” filters out the modules that do not have any activator (BCD, CAD, FKH, DSTAT, ZLD) binding sites (motif score above genomic average). The third column is the number of modules predicted using the selection criteria tabulated in columns 1 and 2. The fourth and fifth columns show the proportion of modules with ChIP support at 1% FDR and 25% FDR, respectively.
(0.03 MB DOC)

Table S5 **Literature evidence of predicted regulatory network edges.** Columns A and C indicate CRM and TF predicted to regulate that CRM; columns D and E indicate ChIP (1% FDR, 25% FDR, or top 100) and DNaseI footprint (FlyReg) support, respectively. Columns F and/or G indicate any literature support for the predicted regulatory interaction, in the form of the PMID (PubMed ID) of the appropriate reference, and “Y” for positive evidence (almost always in the form of expression changes in TF mutant), “N” for no support or negative evidence.
(0.04 MB XLS)

Table S6 **Predicted gene regulatory network of A/P-22 plus FlyExpress genes, mediated by 185 predicted CRMs.**
(0.05 MB XLS)

Table S7 **Regression coefficient of each covariate (and statistical significance of its contribution) when the motif profiles of BCD, CAD, HB, KNI, KR, GT, HKB, and TLL were replaced with corresponding ChIP-on-chip scores, and the model was trained again.** Grayed rows correspond to TFs inferred to be activators (positive coefficient). Note that GT is inferred as an activator despite its well-known role as a repressor. Also note that in comparison to the regression coefficients inferred using motif scores (Table S2A), well-known activators (BCD, CAD, FKH) have a stronger *p* value here, and repressors have a poorer *p* value.
(0.03 MB DOC)

Table S8 **Examples where a CRM has ChIP support for a factor whose expression domain overlaps that of the CRM itself, and which might therefore repress the CRM's activity.** We considered the following expression domains of repressors: GT: bins 20–40 and bins 70–80, KR: bins 40–60, KNI: bins 60–70. CRMs that have expression in a domain where one of the above repressors is expressed and have 1% FDR ChIP support for occupancy by that repressor are listed below. Such listing is shown for known CRMs. “MS-motif-count” is our motif score from multi-species averaging; >0 and <0 indicate above and below the genomic mean, respectively; “significant” indicates >mean + 2 × standard deviation. We examined the ChIP scores profiled through a Genome Browser interface (http://veda.cs.uiuc.edu/lmcrm) to confirm that the ChIP peak is actually centered within the CRM. In case of KNI ChIP, two tracks were considered. For column “Motif Evolution,” we consider the individual motif counts in six species (*D. melanogaster* (mel), *D. ananassae* (ana), *D. pseudoobscura* (pse), *D. virilis* (vir), *D. mojavensis* (moj), *D. grimshawi* (gri)) and note which species have motif count >mean + 2 × standard deviation.
(0.04 MB DOC)

Table S9 **12 predicted CRMs that coincide with known CRMs, and whose experimentally tested activity matches the predicted expression pattern.** *Figure and table references in column 2 refer to graphics within the cited reference (column 3).
(0.03 MB DOC)

Table S10 **Significance assessment of adding additional transcription factors to the original model.** We included BOWL, BTD, NUB, SLP2, and DSTAT (one at a time) in the model to examine if the quality of fit improves. Of these five models evaluated, only the one containing DSTAT shows significant improvement in the quality of fit (see Table 2 for comparison with the original model). However, the model with DSTAT did not improve the sensitivity or specificity of CRM prediction on the “AP-22” set (unpublished data).
(0.03 MB DOC)

Table S11 **The effect of using motif profiles from multiple genomes by two criteria: (A) Agreement between motif profiles and ChIP-on-chip data; shown are the** ***p*** **values of Wilcoxon Rank-Sum test, as in** **Table 1** **, and (B) the quality of model fit using three different goodness of fit measures, as in** **Table 2** **.** (Part of the data in these tables are also shown in Tables 1 and 2.) We note that by criterion (A), there is a significant improvement in going from single species to two species, and also in going from two species to 6 or 11 species comparison, although the latter two are about as effective as each other. By criterion (B), the 6 species motif profiles show the best results (in terms of AIC) followed by 11 species profiles. Considering both criteria, we conclude that while multi-species comparisons clearly improve performance, the advantage of including additional genomes is not as clear in beyond a few genomes.
(0.04 MB DOC)

Table S12 **Genome-wide locations of segments with high pattern generating potential scores.** We segmented the entire genome (after masking for exons) into non-overlapping windows of length 500 bp (“all windows”). The windows with high PGP scores (*p* value <0.0005) for all genes in “A/P-22” set (“high PGP windows”) were examined and compared to all windows, in terms of locations with respect to A/P patterned genes. Each window was considered to be “next to A/P genes” if either its closest neighboring gene is an A/P gene or it is within 10 Kbp of an A/P gene. (The list of A/P genes used here, curated from BDGP images, is shown at http://veda.cs.uiuc.edu/lmcrm.) High PGP scoring windows have 10% chance of being located next to A/P patterned genes, compared to 8% for arbitrary windows (Hypergeometric *p* value 0.0014). In addition, 10% of high PGP windows that are not next to A/P patterned genes have ChIP support at 1% FDR compared to 6% for arbitrary windows not next to A/P patterned genes.
(0.03 MB DOC)

Text S1 **Supplementary notes. (Notes 1, 2, 3, 4, 5, 6, 7, 8, 9, 10.)**
(0.12 MB PDF)

Text S2 **Supplementary methods.**
(0.11 MB PDF)

## Abbreviations

ChIP: Chromatin immunoprecipitation
CRM: *cis*-regulatory module
PGP: pattern generating potential
TF: transcription factor
