# Correction: Quantitative Analysis of the *Drosophila* Segmentation Regulatory Network Using Pattern Generating Potentials

**DOI:** 10.1371/annotation/e38f4ae8-0776-42e6-b912-50800f54436e

**Published:** 2013-10-24

**Authors:** Majid Kazemian, Charles Blatti, Adam Richards, Michael McCutchan, Noriko Wakabayashi-Ito, Ann S. Hammonds, Susan E. Celniker, Sudhir Kumar, Scot A. Wolfe, Michael H. Brodsky, Saurabh Sinha

The original article omitted the complete acknowledgement of the National Institutes of Health's support for the study. Hammonds and Celniker were supported by NIH Grant R01 GM076655 (SEC) through the US Department of Energy under contract no. DE-AC02-05CH11231. 

