# Supplementary figures and images for: Quantitative Analysis of the Drosophila Segmentation Regulatory Network Using Pattern Generating Potentials
Source: PLoS Biol. 2010 Aug 17;8(8):e1000456. doi: 10.1371/journal.pbio.1000456 (PMC2923081; doi:10.1371/journal.pbio.1000456)

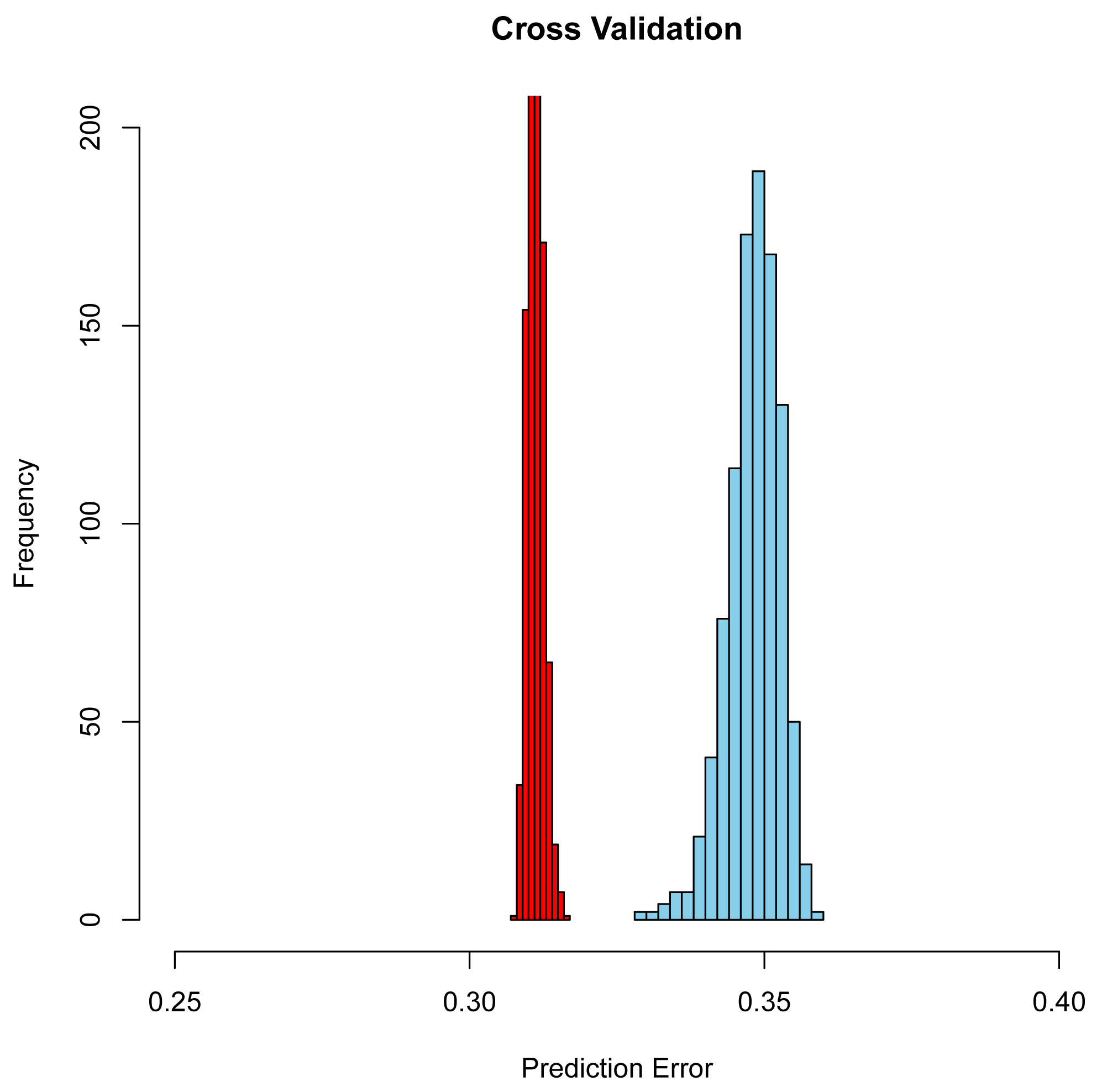

Supplement: Figure S1 — Distribution of prediction error on test data obtained from 1,000 replicates of 2-fold cross-validation, on the original data (red) and randomized data (blue). The cross-validation was done by designating a randomly chosen half of the 4,600 points (46 CRMs×100 bins) as training data and testing the trained model's predictions on the remaining half. Prediction error was defined as the root mean squared error on the test points. A randomized dataset was constructed by randomly permuting the matching between CRMs and their expression profiles. The model is never able to achieve, for the randomized data, the kind of low error rates it achieves on real data (p value = 1.2e-34 based on one-tail Wilcoxon rank-sum). This strongly suggests that the regression model is not “over-fit” to the data of 46 CRMs and their respective expression patterns. (0.17 MB TIF) [file pbio.1000456.s001.tif]

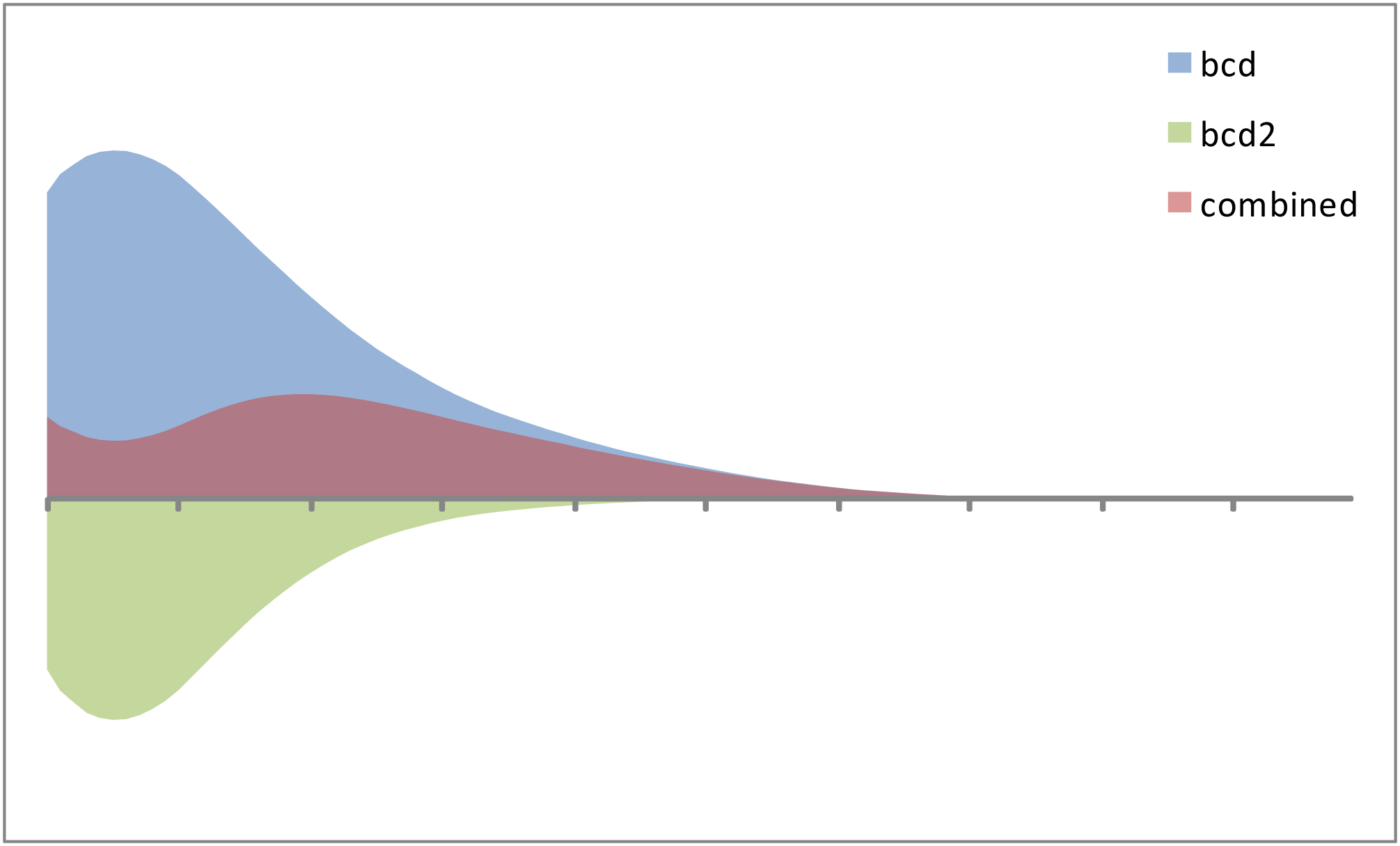

Supplement: Figure S2 — Concentration profile of BCD in our model (with anterior on left). Shown is the effect of BCD, blue, the effect of quadratic form of BCD, green, and the combined effect of both terms, brown, for the CRM btd_head. (0.11 MB TIF) [file pbio.1000456.s002.tif]

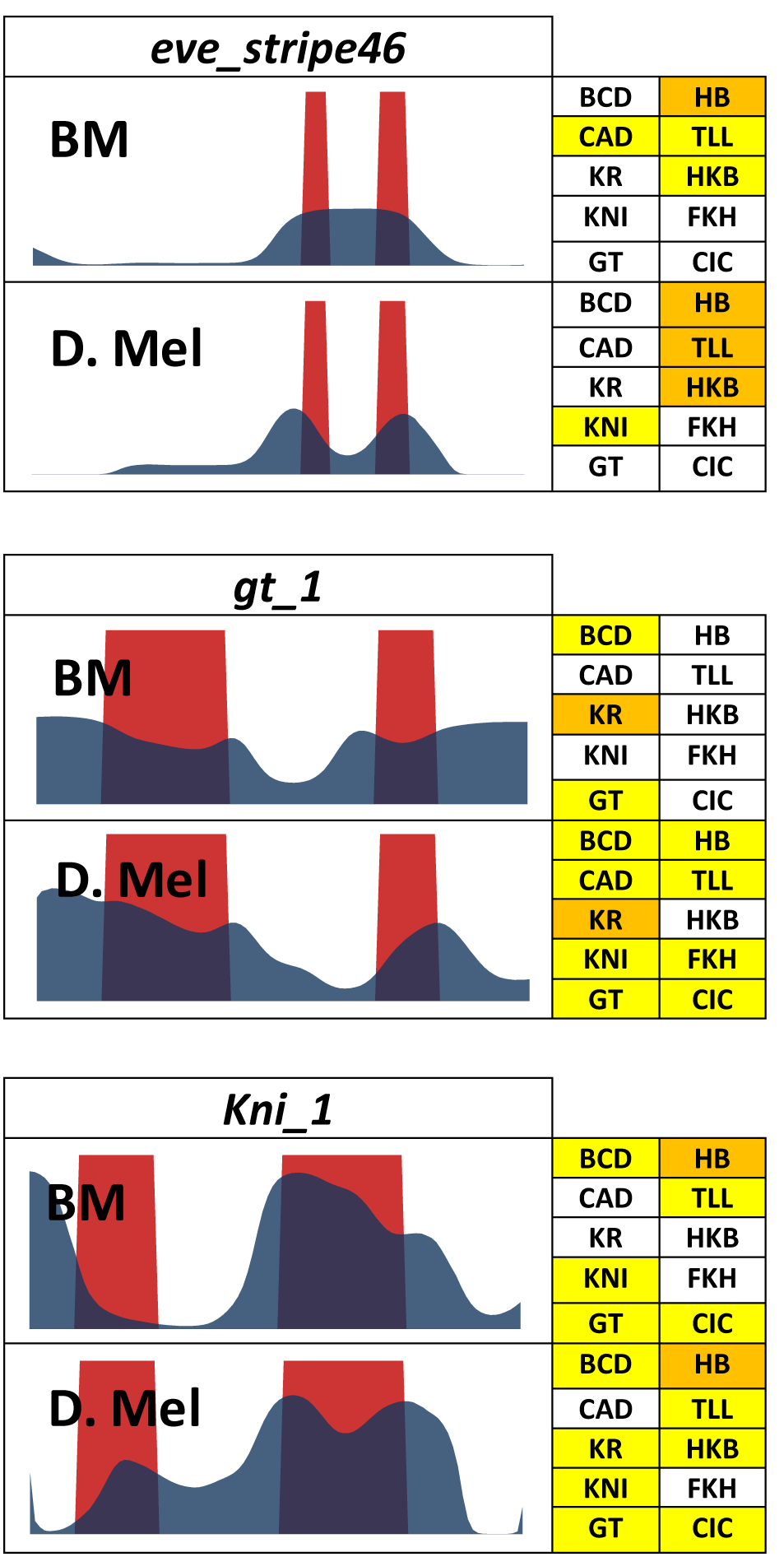

Supplement: Figure S3 — Comparison between predictions with multi-species (BM) and single species ( D. mel ) motif profiles for three modules in which single species perform better than multi-species. The white, yellow, and orange colors represent non-significant, moderate (above genomic mean), and significant (>2 standard deviation above genomic mean) motif counts, respectively. (0.20 MB TIF) [file pbio.1000456.s003.tif]

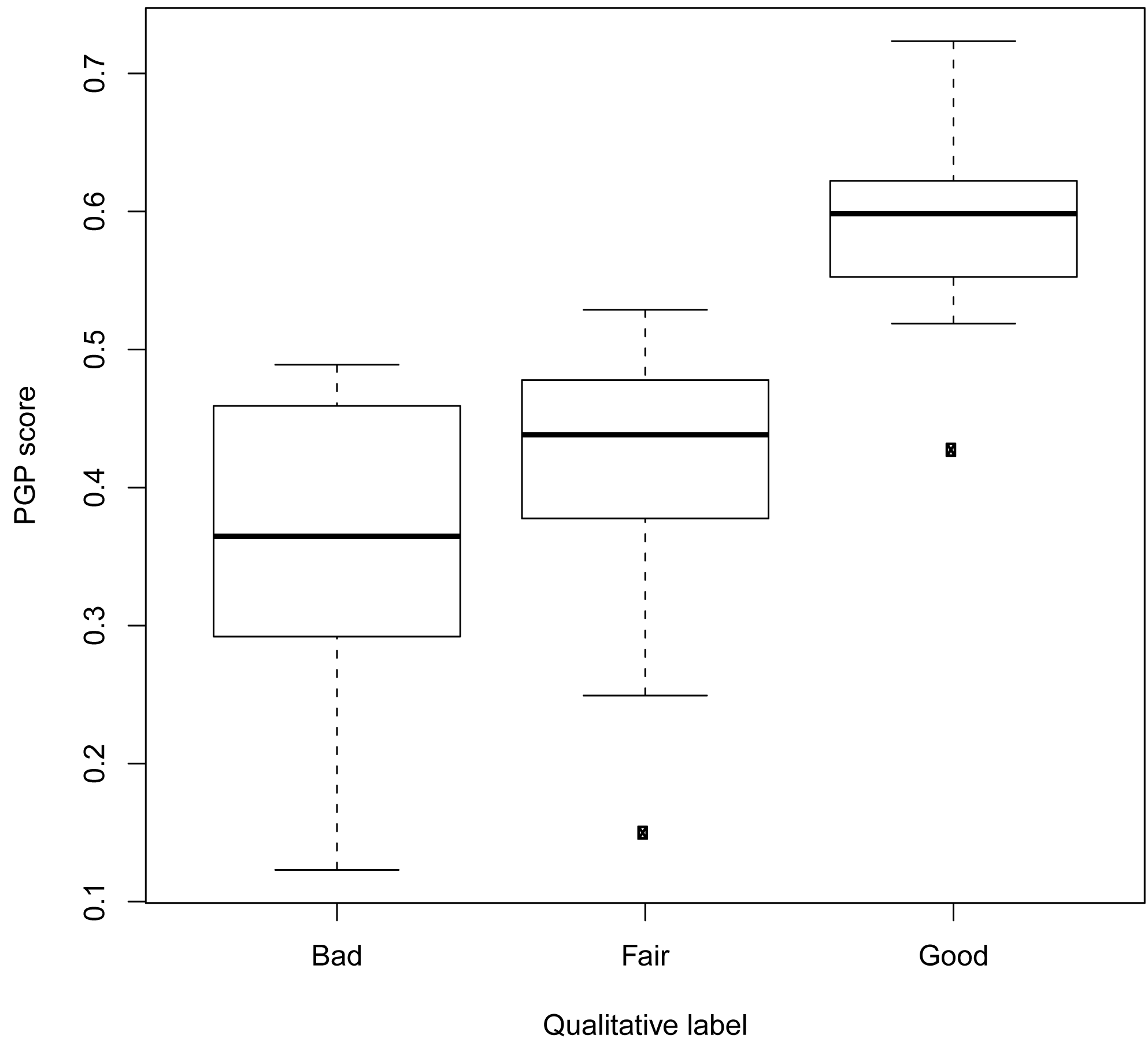

Supplement: Figure S4 — The PGP score, when applied to measure the similarity between predicted and known expression patterns of a CRM, is highly correlated with our visual categorization of the predicted expression as being a “good,” “fair,” or “bad” match to the known expression pattern. These visual categorizations were catalogued in Figure 1B. Shown here is the distribution of PGP scores (means and 25th and 75th quartiles) for each of these three categories, which have 20, 15, and 11 CRMs, respectively. (0.10 MB TIF) [file pbio.1000456.s004.tif]

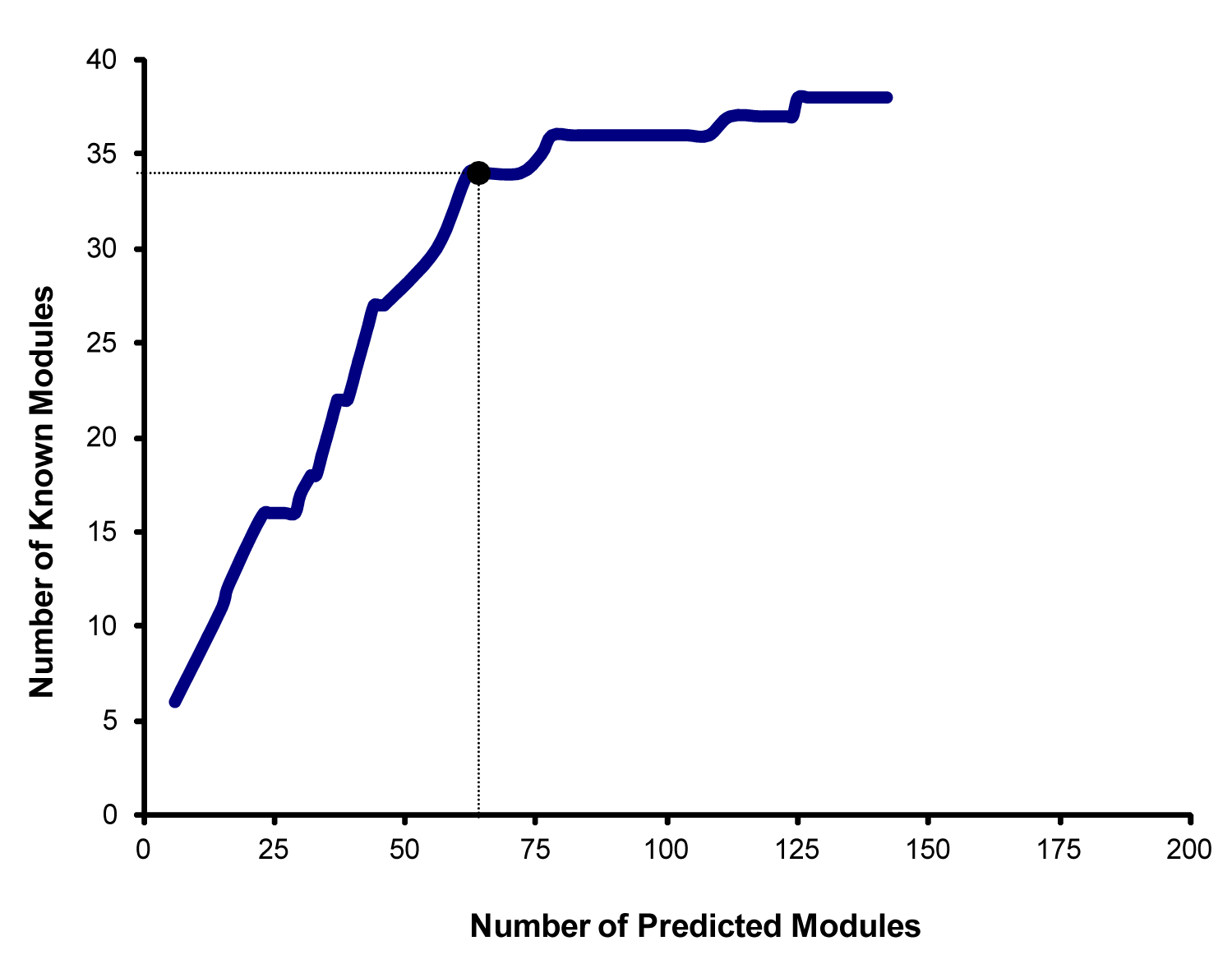

Supplement: Figure S5 — Assessment of the PGP method on A/P-22 set using model trained on all known CRMs. Graph shows the number of retrieved known CRMs (y-axis) as a function of the number of predicted CRMs (x-axis). The dashed line indicates an empirical p value threshold of 0.015, which was used in our final predictions. (0.11 MB TIF) [file pbio.1000456.s005.tif]

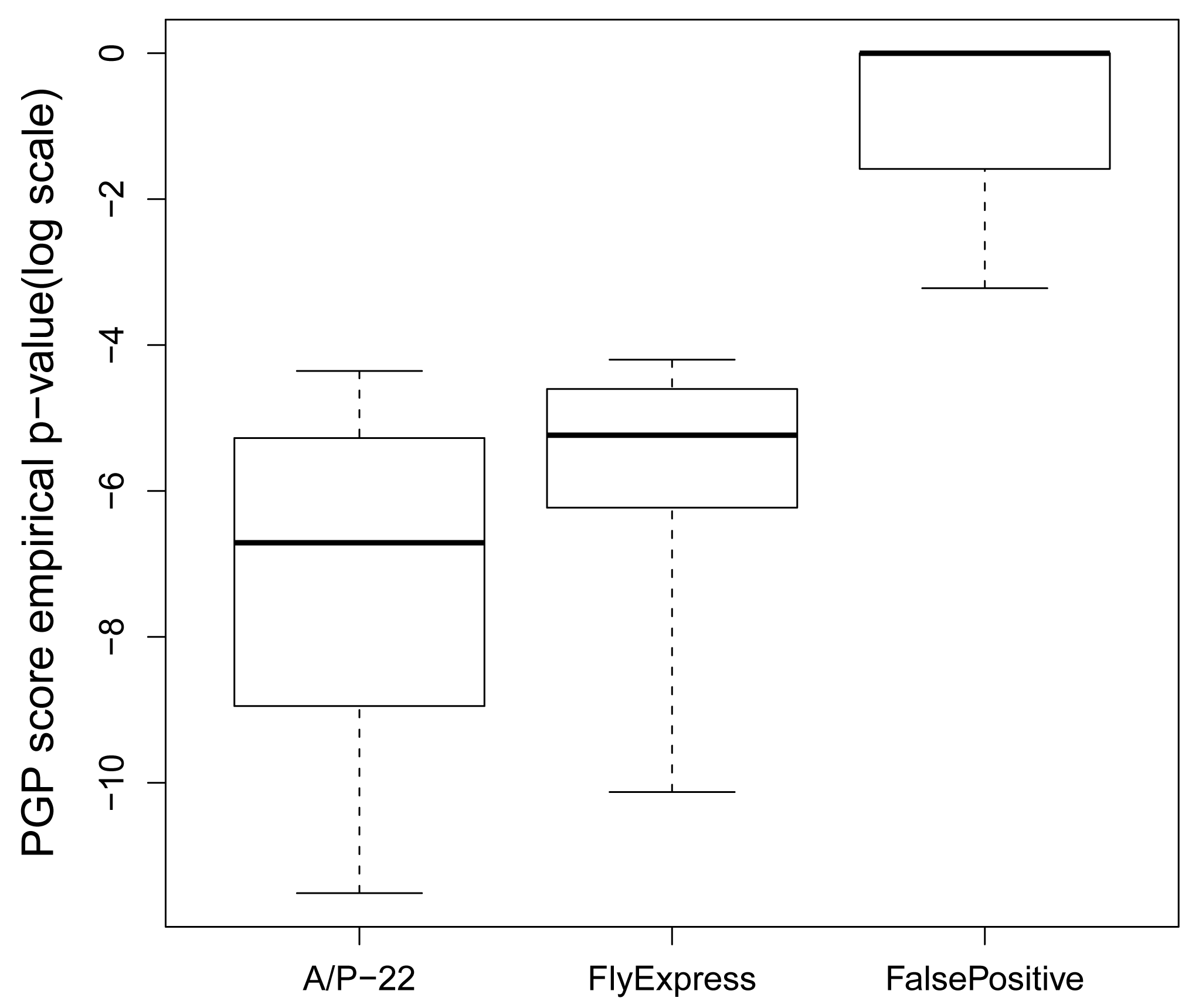

Supplement: Figure S6 — PGP empirical p value for CRMs predicted in the gene sets “A/P-22” (62 CRMs), “FlyExpress” (123 CRMs), as well as the “False Positive” set of eight CRMs. The latter consists of eight experimentally validated CRMs that do not drive any detectable expression in the embryo. These are nub_+5, pdm2_+3, pdm2_+5, and pdm2_+8 from [2] and PCE8008, PCE8021, PCE8023, and PCE8007 from [11]. Additional bona fide CRMs from [11] are not considered because their neighboring genes are not A/P patterned, which implies that those non-CRMs will not even receive a score under our PGP scheme. (0.11 MB TIF) [file pbio.1000456.s006.tif]

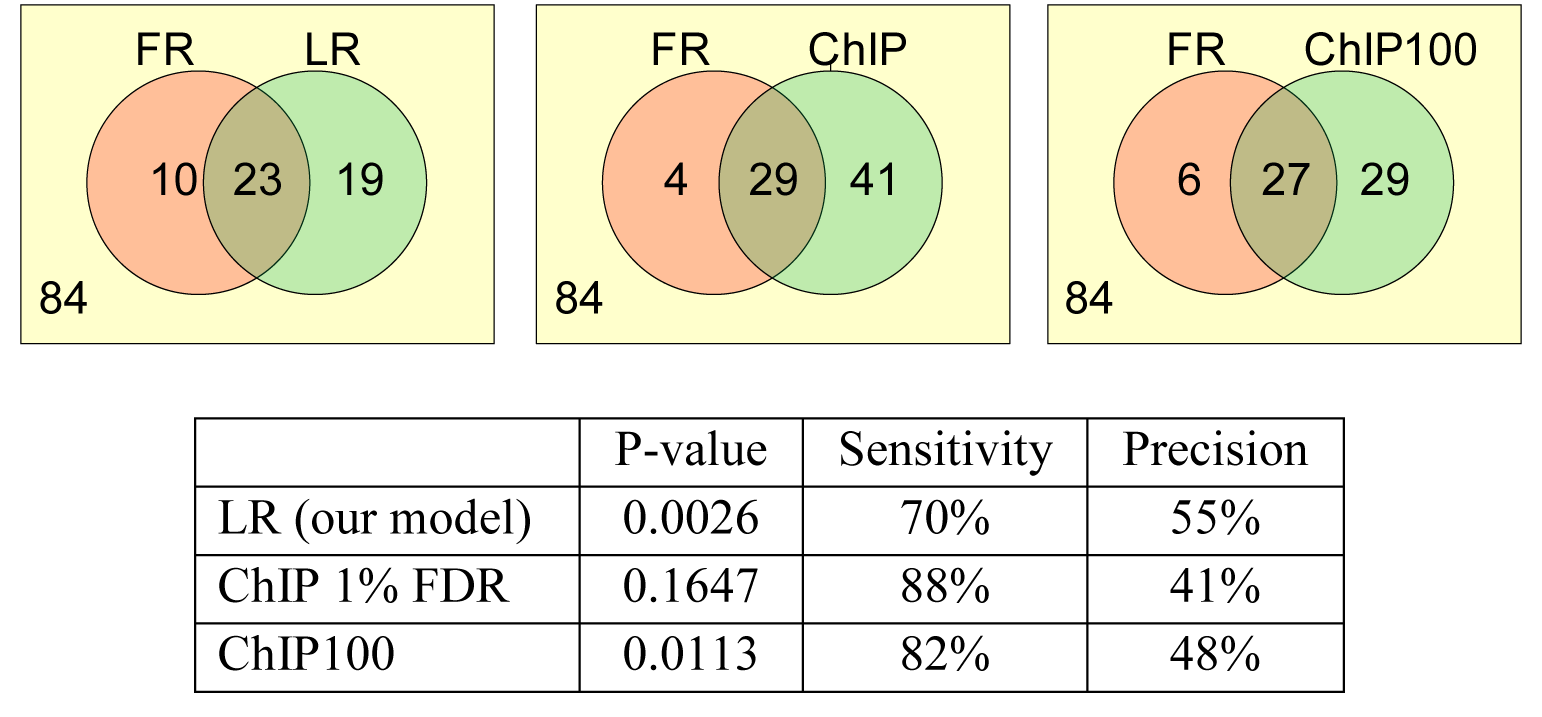

Supplement: Figure S7 — (Top) Overlap between the predicted and experimentally validated regulatory network edges. FR represents 33 experimentally validated edges (from FlyReg) between 12 known CRMs and 7 TFs (BCD, CAD, KR, KNI, HB, GT, and TLL). LR is the set of regulatory edges predicted by our linear regression model for the same set of CRMs and factors. “ChIP” refers to edges inferred based on TF occupancy revealed by ChIP-chip (at 1% FDR). “ChIP100” is the same dataset, except that only the top 100 bound regions are considered. The total number of possible edges is 12×7 = 84. (Bottom) Sensitivity, precision, and Hypergeometric p values of overlap of each method's edge predictions with the “test” regulatory network comprising 33 experimentally validated edges from FlyReg. (0.15 MB TIF) [file pbio.1000456.s007.tif]

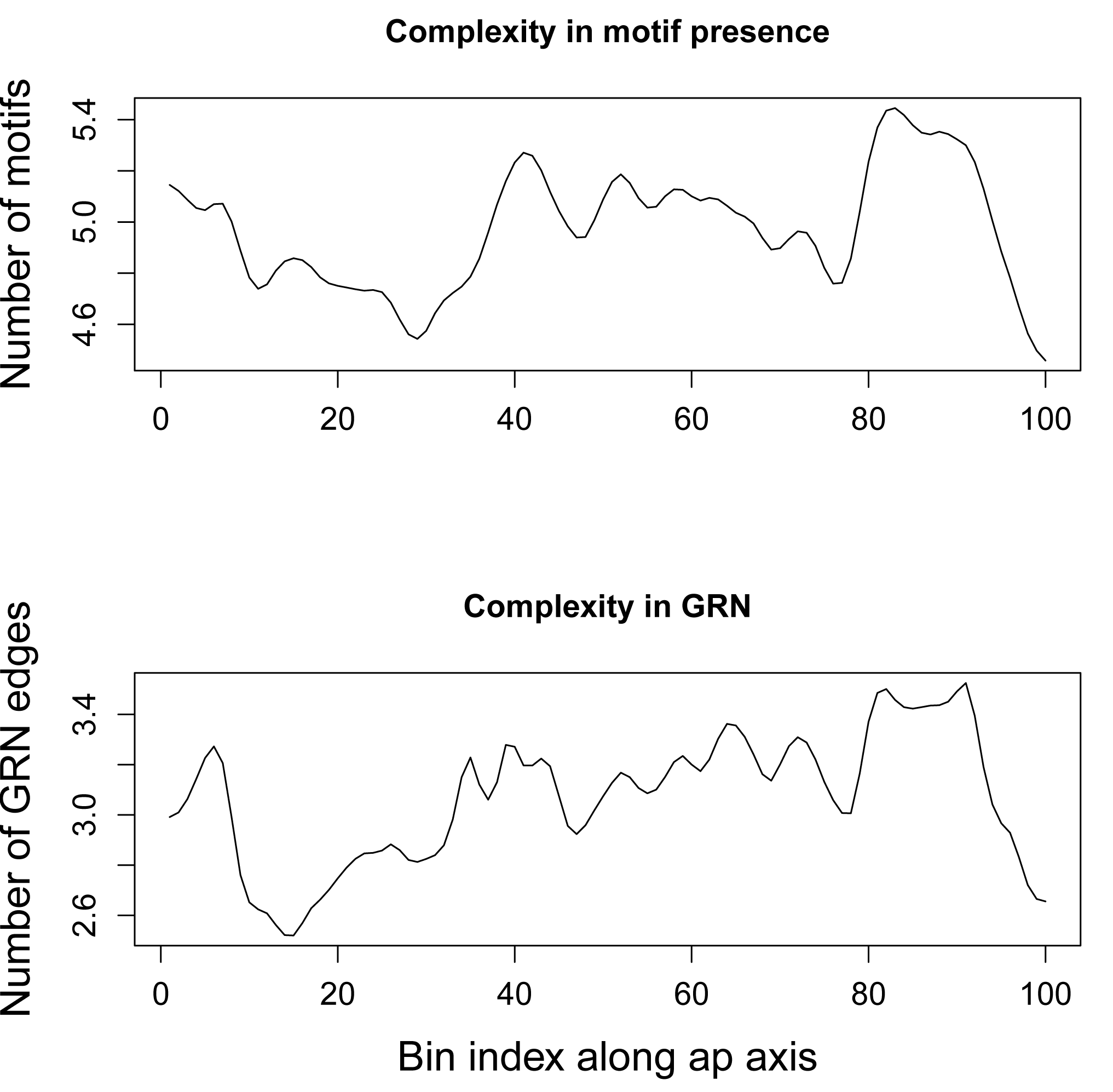

Supplement: Figure S8 — (Top) Distribution of the average number of motifs present (motif score >0) in all predicted CRMs driving expression in a bin along the A/P axis. (Bottom) Average number of motifs (TFs) with “regulatory edges” to all predicted CRMs driving expression in a particular bin along the A/P axis. (0.17 MB TIF) [file pbio.1000456.s008.tif]

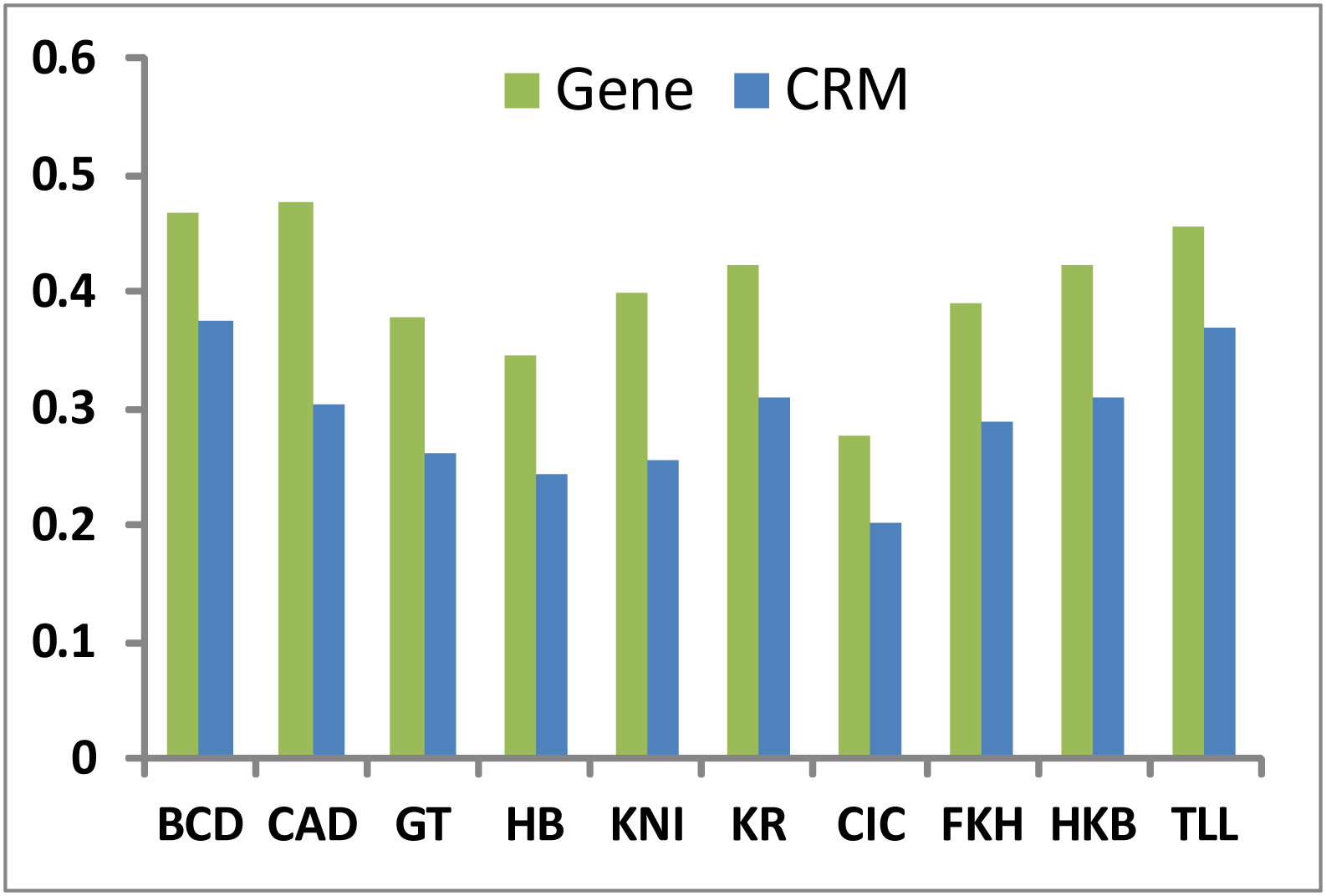

Supplement: Figure S9 — Shown is the fraction of genes/CRMs targeted by each transcription factor (as per our regulatory edge prediction method) in the A/P-22 and FlyExpress gene sets. The number of interactions with genes/CRMs is almost uniform across TFs. (0.15 MB TIF) [file pbio.1000456.s009.tif]

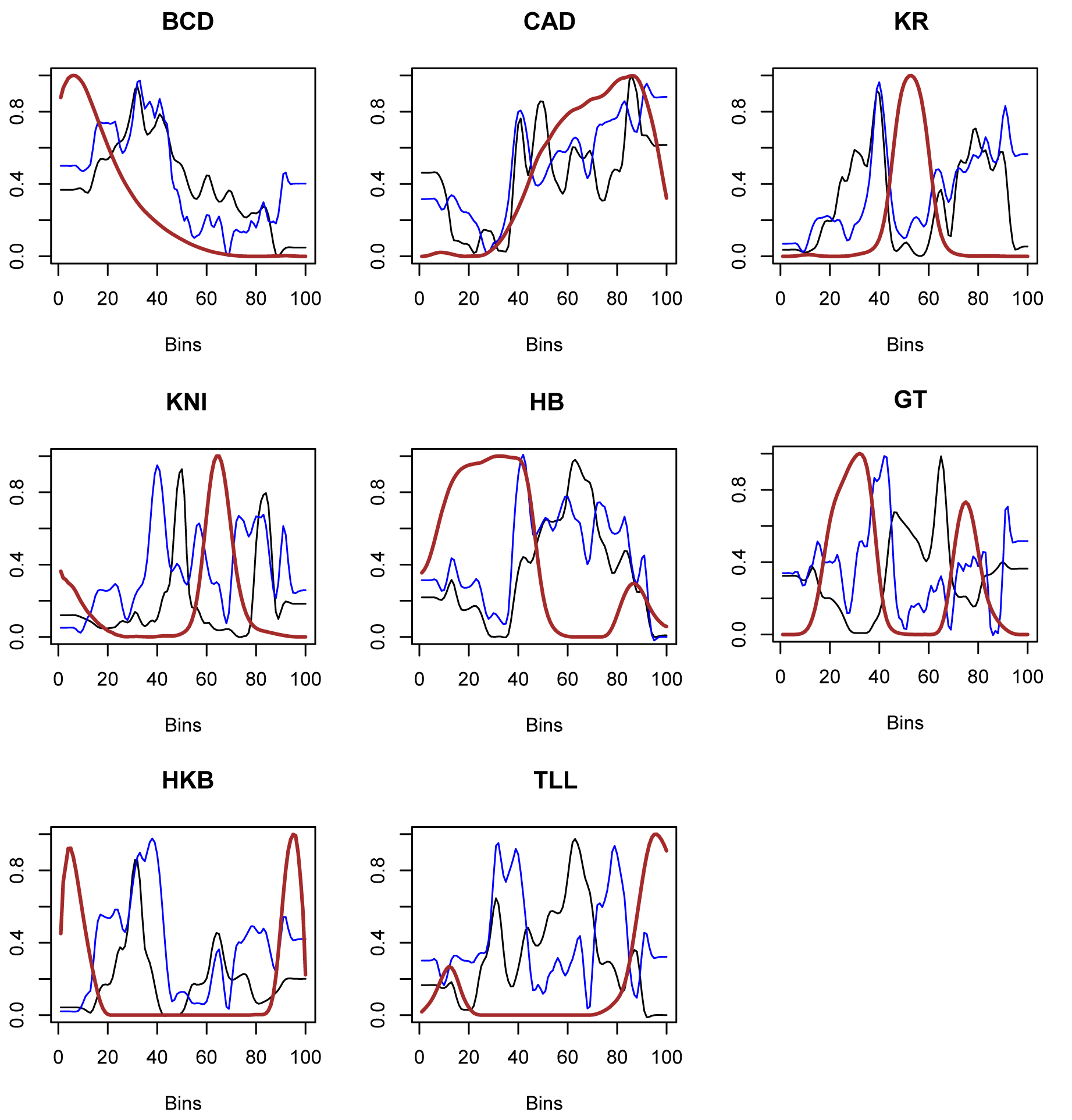

Supplement: Figure S10 — The contribution of individual transcription factors to the 46 known CRMs, as a function of the position along the A/P axis where the CRMs drive expression. For each position (x-axis), shown are the average motif score (black) and ChIP-chip score (blue) of the factor in CRMs driving expression at that position. The red curve is the concentration profile of the transcription factor. Note that for KNI and GT, the black curve (motif score based) shows much better anti-correlation with the TF profile than does the blue curve (ChIP based). (0.32 MB TIF) [file pbio.1000456.s010.tif]

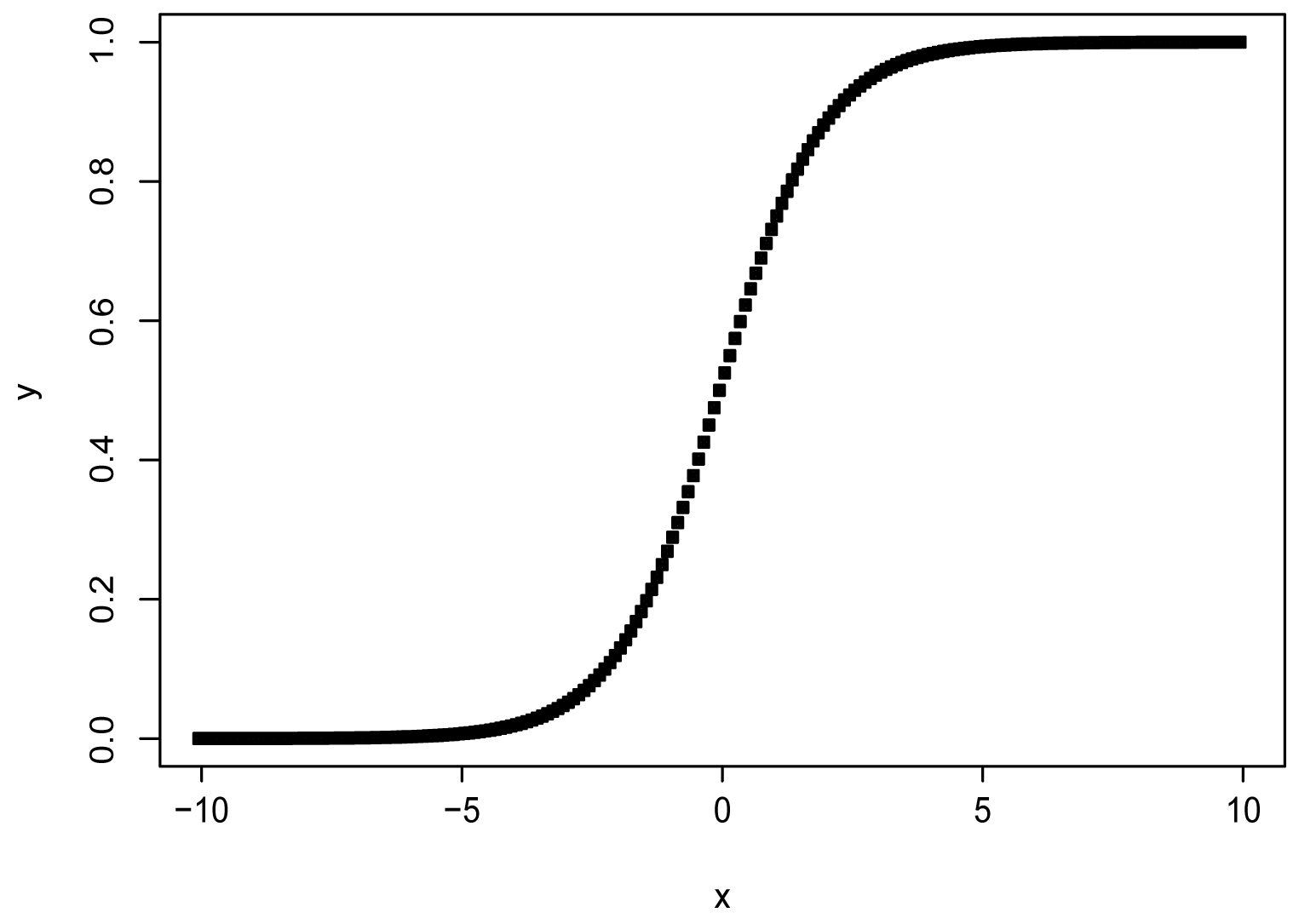

Supplement: Figure S11 — The logistic function used in our logistic regression model. The logistic function is a commonly used S-shaped function that takes values between 0 and 1. The logistic function used here is y = 1/(1+exp(−x)). (0.07 MB TIF) [file pbio.1000456.s011.tif]
