# Supplementary material for: Quantitative Analysis of the Drosophila Segmentation Regulatory Network Using Pattern Generating Potentials
Source: PLoS Biol. 2010 Aug 17;8(8):e1000456. doi: 10.1371/journal.pbio.1000456 (PMC2923081; doi:10.1371/journal.pbio.1000456)
Supplement: Table S1 — The first row summarizes the visual classification of the quality of fit (as “good,” “fair,” or “bad”) between the model's prediction and known expression patterns of 46 known CRMs. The second row shows the results of a similar visual classification of predictions from a previous thermodynamic model of [20]. These results should not be interpreted as a strict comparison of models, since the numbers and identities of CRMs used in the two analyses are slightly different, the numbers of motifs used as input are different, and the numbers of free parameters trained by the models are widely different. Moreover, the predictions from the Segal et al. model are not publicly available in a machine-readable format. (0.03 MB DOC) [file pbio.1000456.s012.doc]

|  | ***Good*** | ***Fair*** | ***Bad*** | ***Total*** |
| --- | --- | --- | --- | --- |
| **Logistic Regression Model** | 20 | 15 | 11 | 46 |
| **Thermodynamic Model** | 17 | 18 | 9 | 44 |
