# Supplementary material for: Quantitative Analysis of the Drosophila Segmentation Regulatory Network Using Pattern Generating Potentials
Source: PLoS Biol. 2010 Aug 17;8(8):e1000456. doi: 10.1371/journal.pbio.1000456 (PMC2923081; doi:10.1371/journal.pbio.1000456)
Supplement: Table S2 — (A) Covariates of our model and the significance of their contributions: the first column lists the model covariates (predictors). The second column is the trained value of the regression coefficient (wi) of each covariate, and the third column is the significance of the coefficients (computed as described in Methods). Grayed rows correspond to TFs inferred to be activators (positive coefficients), while others are repressors (negative coefficients). (B) The trained value of the “baseline” parameter for each CRM: the baseline values range from ∼−4.5 to ∼3 with an average of −1.4 (0.06 MB DOC) [file pbio.1000456.s013.doc]

**A**

| ***Covariate*** | ***Coefficient*** | ***p-value*** |
| --- | --- | --- |
| **BCD** | 0.051 | 3.3E-44 |
| **CAD** | 0.034 | 2.5E-10 |
| **HB** | -0.012 | 6.6E-26 |
| **KNI** | -0.027 | 1.0E-11 |
| **KR** | -0.0095 | 1.5E-10 |
| **GT** | -0.032 | 2.1E-14 |
| **TLL** | -0.042 | 1.2E-20 |
| **FKH** | 0.028 | 3.2E-06 |
| **CIC** | -3.4 | 5.1E-12 |
| **HKB** | -0.044 | 9.8E-08 |
| **BCD2** | -0.00019 | 2.6E-26 |

**B**

| ***Covariate*** | ***Coefficient*** | ***p-value*** | ***Covariate*** | ***Coefficient*** | ***p-value*** |
| --- | --- | --- | --- | --- | --- |
| **btd_head** | -3.608 | 5.2E-19 | **hb_anterior_actv** | -1.760 | 1.9E-10 |
| **cad_14** | -4.299 | 9.5E-12 | **hb_centr_and_post** | -0.842 | 2.7E-02 |
| **cnc_5** | -2.507 | 1.3E-12 | **hkb_ventral_elem** | -1.220 | 3.7E-02 |
| **D_4** | 0.126 | 7.1E-01 | **kni_1** | 2.998 | 5.2E-19 |
| **ems_head** | -3.558 | 3.4E-16 | **kni_5** | -0.569 | 1.3E-01 |
| **eve_stripe1** | -4.144 | 9.1E-14 | **kni_kd** | -0.248 | 6.5E-01 |
| **eve_stripe2** | -3.904 | 1.4E-15 | **knrl_8** | -0.470 | 7.0E-02 |
| **eve_stripe3_7** | -0.374 | 4.5E-01 | **Kr_AD2** | 0.073 | 8.3E-01 |
| **eve_stripe4_6** | -0.814 | 2.1E-02 | **Kr_CD1** | -1.577 | 2.8E-07 |
| **eve_stripe5** | -0.023 | 9.7E-01 | **Kr_CD2_AD1** | 2.650 | 3.8E-11 |
| **fkh_2** | -3.425 | 3.9E-12 | **nub_2** | -0.199 | 4.1E-01 |
| **ftz_ps4_actv** | -2.067 | 9.8E-06 | **oc_7** | -2.948 | 1.8E-20 |
| **gt_1** | -0.109 | 6.2E-01 | **oc_otd_early** | -1.971 | 7.7E-13 |
| **gt_10** | -1.743 | 1.2E-06 | **odd_3** | -0.912 | 2.7E-02 |
| **gt_3** | -1.133 | 3.1E-03 | **odd_5** | -1.653 | 3.0E-07 |
| **gt_6** | -3.319 | 1.2E-17 | **pdm2_1** | 0.258 | 3.9E-01 |
| **gt_berman** | -1.034 | 3.1E-03 | **run_stripe1** | -3.817 | 1.5E-12 |
| **h_stripe1** | -3.796 | 9.1E-15 | **run_stripe3** | -1.857 | 1.3E-03 |
| **h_stripe3** | -2.015 | 6.0E-05 | **run_stripe5** | -2.133 | 3.5E-04 |
| **h_stripe4** | -1.638 | 2.9E-03 | **slp2_3** | -2.720 | 1.8E-13 |
| **h_stripe5** | 0.677 | 2.7E-01 | **tll_K2** | 0.295 | 3.8E-01 |
| **h_stripe6** | -3.005 | 4.9E-09 | **tll_P2** | -0.036 | 8.9E-01 |
| **h_stripe7** | -1.441 | 6.4E-03 | **tll_P3** | 0.415 | 2.6E-01 |
