# Supplementary material for: Quantitative Analysis of the Drosophila Segmentation Regulatory Network Using Pattern Generating Potentials
Source: PLoS Biol. 2010 Aug 17;8(8):e1000456. doi: 10.1371/journal.pbio.1000456 (PMC2923081; doi:10.1371/journal.pbio.1000456)
Supplement: Table S3 — Statistical evidence for prevalence of functionally redundant (“sibling”) CRMs near maternal and gap genes. CRMs driving a particular aspect of a target gene G's expression pattern were predicted genome-wide using the PGP method. These CRMs may be located in the control region of gene G itself, or not. Those located in the control region of the target gene itself are called “real” and the rest are called “random.” The one or more predicted CRMs in the control region of the same gene (which may or may not be the target gene), driving the same expression pattern, are defined as a CRM set. A CRM set may be “solitary” (cardinality of one) or “redundant” (cardinality of more than one). The predicted CRMs constituting a redundant CRM set are functionally redundant CRMs, potentially. Also, as noted above, a CRM set may be “real” (if located in the control region of the target gene) or “random.” A 2×2 contingency table is thus defined and its statistical significance estimated by the Fisher's exact test. The p value obtained for this table is 4.0E-4, strongly suggesting that “real” CRMs (i.e., predicted CRMs more likely to be true positives) are enriched for the property of having functionally related partners (“siblings”). (0.03 MB DOC) [file pbio.1000456.s014.doc]

|  | ***Real*** | ***Random*** |
| --- | --- | --- |
| **Redundant** | 15 | 11171 |
| **Solitary** | 7 | 24506 |
