# Supplementary material for: Quantitative Analysis of the Drosophila Segmentation Regulatory Network Using Pattern Generating Potentials
Source: PLoS Biol. 2010 Aug 17;8(8):e1000456. doi: 10.1371/journal.pbio.1000456 (PMC2923081; doi:10.1371/journal.pbio.1000456)
Supplement: Table S4 — Properties of FlyExpress predicted modules, shown for various selection criteria. The grayed row corresponds to the criteria used in the paper. The first column shows the PGP p value threshold used in prediction of modules. The second column lists the type of additional filter applied for selecting modules: “Best of each gene” indicates that only the module with the lowest p value for the gene was selected. “Activator presence” filters out the modules that do not have any activator (BCD, CAD, FKH, DSTAT, ZLD) binding sites (motif score above genomic average). The third column is the number of modules predicted using the selection criteria tabulated in columns 1 and 2. The fourth and fifth columns show the proportion of modules with ChIP support at 1% FDR and 25% FDR, respectively. (0.03 MB DOC) [file pbio.1000456.s015.doc]

| ***p-value*** | ***Filter Type*** | ***#Predictions*** | ***1%ChIP*** | ***25%ChIP*** |
| --- | --- | --- | --- | --- |
| 0.015 | none | 132 | 0.417 | 0.644 |
| 0.015 | best of each gene | 71 | 0.493 | 0.690 |
| 0.015 | activator presence | 123 | 0.439 | 0.650 |
| 0.03 | none | 210 | 0.348 | 0.619 |
| 0.03 | best of each gene | 88 | 0.420 | 0.625 |
| 0.03 | activator presence | 193 | 0.368 | 0.632 |
