# Supplementary material for: Quantitative Analysis of the Drosophila Segmentation Regulatory Network Using Pattern Generating Potentials
Source: PLoS Biol. 2010 Aug 17;8(8):e1000456. doi: 10.1371/journal.pbio.1000456 (PMC2923081; doi:10.1371/journal.pbio.1000456)
Supplement: Table S7 — Regression coefficient of each covariate (and statistical significance of its contribution) when the motif profiles of BCD, CAD, HB, KNI, KR, GT, HKB, and TLL were replaced with corresponding ChIP-on-chip scores, and the model was trained again. Grayed rows correspond to TFs inferred to be activators (positive coefficient). Note that GT is inferred as an activator despite its well-known role as a repressor. Also note that in comparison to the regression coefficients inferred using motif scores (Table S2A), well-known activators (BCD, CAD, FKH) have a stronger p value here, and repressors have a poorer p value. (0.03 MB DOC) [file pbio.1000456.s018.doc]

| ***Covariate*** | ***Coefficient*** | ***p-value*** |
| --- | --- | --- |
| **BCD** | 0.0558 | 2.5E-50 |
| **CAD** | 0.0175 | 3.0E-30 |
| **HB** | -0.0019 | 1.1E-02 |
| **KNI** | -0.0032 | 3.5E-03 |
| **KR** | -0.0002 | 7.6E-01 |
| **GT** | 0.0011 | 7.1E-02 |
| **TLL** | -0.0052 | 2.0E-04 |
| **FKH** | 0.0492 | 3.9E-13 |
| **CIC** | -6.6542 | 8.6E-23 |
| **HKB** | -0.0129 | 2.5E-14 |
| **BCD2** | -0.0001 | 6.5 E-24 |
