# Supplementary material for: Quantitative Analysis of the Drosophila Segmentation Regulatory Network Using Pattern Generating Potentials
Source: PLoS Biol. 2010 Aug 17;8(8):e1000456. doi: 10.1371/journal.pbio.1000456 (PMC2923081; doi:10.1371/journal.pbio.1000456)
Supplement: Table S8 — Examples where a CRM has ChIP support for a factor whose expression domain overlaps that of the CRM itself, and which might therefore repress the CRM's activity. We considered the following expression domains of repressors: GT: bins 20–40 and bins 70–80, KR: bins 40–60, KNI: bins 60–70. CRMs that have expression in a domain where one of the above repressors is expressed and have 1% FDR ChIP support for occupancy by that repressor are listed below. Such listing is shown for known CRMs. “MS-motif-count” is our motif score from multi-species averaging; >0 and <0 indicate above and below the genomic mean, respectively; “significant” indicates >mean + 2 × standard deviation. We examined the ChIP scores profiled through a Genome Browser interface (http://veda.cs.uiuc.edu/lmcrm) to confirm that the ChIP peak is actually centered within the CRM. In case of KNI ChIP, two tracks were considered. For column “Motif Evolution,” we consider the individual motif counts in six species (D. melanogaster (mel), D. ananassae (ana), D. pseudoobscura (pse), D. virilis (vir), D. mojavensis (moj), D. grimshawi (gri)) and note which species have motif count >mean + 2 × standard deviation. (0.04 MB DOC) [file pbio.1000456.s019.doc]

| ***CRM name*** | ***Factor*** | ***MS-motif-count*** | ***Motif Evolution*** |
| --- | --- | --- | --- |
| cnc_5 | GT | >0 | *mel, ana, pse* |
| D_4 | KR | significant | *mel, ana, pse, vir, moj, gri* |
| D_4 | KNI | <0 | *gri* |
| D_4 | GT | >0 | *gri, moj* |
| gt_10 | GT | <0 | NONE |
| gt_3 | GT | >0 | *mel, ana* |
| gt_berman | GT | >0 | *mel, ana* |
| h_stripe1 | GT | <0 | NONE |
| h_stripe6 | GT | <0 | *gri* |
| hb_anterior | GT | <0 | *gri* |
| kni_kd | KNI | >0 | *mel, ana* |
| kr_CD1 | KR | >0 | *moj* |
| kr_CD2_AD1 | KR | significant | *mel, ana, pse, vir, moj, gri* |
| nub_2 | GT | <0 | *mel* |
| oc_7 | GT | <0 | *pse* |
| oc_otd_early | GT | <0 | NONE |
| odd_5 | GT | <0 | NONE |
| run_stripe1 | GT | <0 | NONE |
| slp2_3 | GT | <0 | NONE |
