# Supplementary material for: Quantitative Analysis of the Drosophila Segmentation Regulatory Network Using Pattern Generating Potentials
Source: PLoS Biol. 2010 Aug 17;8(8):e1000456. doi: 10.1371/journal.pbio.1000456 (PMC2923081; doi:10.1371/journal.pbio.1000456)
Supplement: Table S9 — 12 predicted CRMs that coincide with known CRMs, and whose experimentally tested activity matches the predicted expression pattern. *Figure and table references in column 2 refer to graphics within the cited reference (column 3). (0.03 MB DOC) [file pbio.1000456.s020.doc]

| ***Predicted CRM*** | ***Literature constructs with matching activity^*^*** | ***Reference (PMID)*** |
| --- | --- | --- |
| En.1 | en_upstream_enhancer; A multipartite PRE near the en transcription start site (from -2.4 kb to -395 bp) | [18199580](http://dev.biologists.org/content/135/4/669.long) |
| Prd.1 | prd_Pstripe_enhancer; prdDelta(I), prdDelta(L), Figure 4E(sb) | [7873402](http://www.ncbi.nlm.nih.gov/pubmed/7873402) |
| Prd.2 | Prd_O-E; (Figure 2J, Table 2) | [15793007](http://www.pnas.org/content/102/14/4960.long) |
| Slp1.1 | Slp1_slp_A; (Figure 2L, Table 2) | [15793007](http://www.pnas.org/content/102/14/4960.long) |
| Slp1.2 | Slp2_(-3); (Figure 2g) | [15340490](http://www.plosbiology.org/article/info%3Adoi%2F10.1371%2Fjournal.pbio.0020271) |
| Slp1.3 | Slp1_slp_B; (Figure 2M, Table 2) | [15793007](http://www.pnas.org/content/102/14/4960.long) |
| Stg.1 | stg_bE49; pstgBeta-E4.9 (Figure 2E) | [10101114](http://dev.biologists.org/content/126/9/1793.long) |
| Gcm.1 | gcm_49;Figure 2 | [14738884](http://www.ncbi.nlm.nih.gov/pubmed/14738884) |
| Gcm.2 | gcm_7444; Figure 4 | [14738884](http://www.ncbi.nlm.nih.gov/pubmed/14738884) |
| Srp.1 | Srp:5utr; (Figure 2G) | [19853570](http://www.sciencedirect.com/science?_ob=ArticleURL&_udi=B6WW3-4XGYBVP-J&_user=571676&_rdoc=1&_fmt=&_orig=search&_sort=d&_docanchor=&view=c&_acct=C000029040&_version=1&_urlVersion=0&_userid=571676&md5=5d1330f386b58f6d9cd5e6f04a3d7be9) |
| CG31670.1 | Figure 3B | [18621688](http://www.ncbi.nlm.nih.gov/pubmed/18621688) |
| CG31670.2 | Figure 3B | [18621688](http://www.ncbi.nlm.nih.gov/pubmed/18621688) |
