# Supplementary material for: Quantitative Analysis of the Drosophila Segmentation Regulatory Network Using Pattern Generating Potentials
Source: PLoS Biol. 2010 Aug 17;8(8):e1000456. doi: 10.1371/journal.pbio.1000456 (PMC2923081; doi:10.1371/journal.pbio.1000456)
Supplement: Table S10 — Significance assessment of adding additional transcription factors to the original model. We included BOWL, BTD, NUB, SLP2, and DSTAT (one at a time) in the model to examine if the quality of fit improves. Of these five models evaluated, only the one containing DSTAT shows significant improvement in the quality of fit (see Table 2 for comparison with the original model). However, the model with DSTAT did not improve the sensitivity or specificity of CRM prediction on the “AP-22” set (unpublished data). (0.03 MB DOC) [file pbio.1000456.s021.doc]

| ***Model*** | ***ACC*** | ***RMSE*** | ***AIC*** | ***Coefficient*** | ***p-value*** |
| --- | --- | --- | --- | --- | --- |
| ***BOWL*** | 0.482 | 0.305 | 2895 | 0.013 | 2.67E-001 |
| ***BTD*** | 0.485 | 0.305 | 2895 | 0.006 | 4.01E-001 |
| ***NUB*** | 0.483 | 0.305 | 2895 | 0.009 | 2.20E-001 |
| ***SLP2*** | 0.482 | 0.305 | 2895 | -0.009 | 3.53E-001 |
| ***DSTAT*** | 0.526 | 0.301 | 2819 | 0.016 | 1.99E-013 |
