# Supplementary material for: Quantitative Analysis of the Drosophila Segmentation Regulatory Network Using Pattern Generating Potentials
Source: PLoS Biol. 2010 Aug 17;8(8):e1000456. doi: 10.1371/journal.pbio.1000456 (PMC2923081; doi:10.1371/journal.pbio.1000456)
Supplement: Table S11 — The effect of using motif profiles from multiple genomes by two criteria: (A) Agreement between motif profiles and ChIP-on-chip data; shown are the p values of Wilcoxon Rank-Sum test, as in Table 1 , and (B) the quality of model fit using three different goodness of fit measures, as in Table 2 . (Part of the data in these tables are also shown in Tables 1 and 2.) We note that by criterion (A), there is a significant improvement in going from single species to two species, and also in going from two species to 6 or 11 species comparison, although the latter two are about as effective as each other. By criterion (B), the 6 species motif profiles show the best results (in terms of AIC) followed by 11 species profiles. Considering both criteria, we conclude that while multi-species comparisons clearly improve performance, the advantage of including additional genomes is not as clear in beyond a few genomes. (0.04 MB DOC) [file pbio.1000456.s022.doc]

A

| ***TF*** | ***Single Species*** | ***2 species*** | ***6 species*** | ***11 species*** |
| --- | --- | --- | --- | --- |
| BCD | 2.0E-25 | 2.0E-33 | 1.7E-44 | 1.6E-45 |
| CAD | 1.5E-5 | 1.5E-15 | 3.3E-23 | 1.9E-27 |
| GT | 8.3E-7 | 8.5E-11 | 9.7E-17 | 2.3E-22 |
| HB | 1.8E-3 | 3.3E-5 | 5.3E-12 | 7.0E-15 |
| KNI | 1.8E-4 | 2.3E-5 | 1.1E-17 | 7.5E-10 |
| KR | 2.6E-13 | 3.5E-17 | 1.8E-26 | 3.6E-31 |
| HKB | 5.7E-23 | 8.7E-23 | 5.9E-34 | 7.4E-35 |
| TLL | 2.0E-4 | 1.3E-8 | 4.1E-19 | 3.1E-20 |

B

| ***Model Implementation*** | ***RMSE*** | ***ACC*** | ***AIC*** |
| --- | --- | --- | --- |
| Single Species | 0.309 | 0.46 | 2962 |
| 2 Species (*mel, pse*) | 0.308 | 0.47 | 2959 |
| 6 Species (*mel, ana, pse, vir, moj, gri*) | 0.302 | 0.49 | 2843 |
| 11 Species | 0.305 | 0.48 | 2894 |
