# Supplementary material for: Quantitative Analysis of the Drosophila Segmentation Regulatory Network Using Pattern Generating Potentials
Source: PLoS Biol. 2010 Aug 17;8(8):e1000456. doi: 10.1371/journal.pbio.1000456 (PMC2923081; doi:10.1371/journal.pbio.1000456)
Supplement: Table S12 — Genome-wide locations of segments with high pattern generating potential scores. We segmented the entire genome (after masking for exons) into non-overlapping windows of length 500 bp (“all windows”). The windows with high PGP scores (p value <0.0005) for all genes in “A/P-22” set (“high PGP windows”) were examined and compared to all windows, in terms of locations with respect to A/P patterned genes. Each window was considered to be “next to A/P genes” if either its closest neighboring gene is an A/P gene or it is within 10 Kbp of an A/P gene. (The list of A/P genes used here, curated from BDGP images, is shown at http://veda.cs.uiuc.edu/lmcrm.) High PGP scoring windows have 10% chance of being located next to A/P patterned genes, compared to 8% for arbitrary windows (Hypergeometric p value 0.0014). In addition, 10% of high PGP windows that are not next to A/P patterned genes have ChIP support at 1% FDR compared to 6% for arbitrary windows not next to A/P patterned genes. (0.03 MB DOC) [file pbio.1000456.s023.doc]

|  | ***Location*** | ***Frequency*** | ***1%ChIP*** |
| --- | --- | --- | --- |
| **all windows** | anywhere | 177502 | 8% |
|  | next to A/P genes | 14277 (8%) | 22% |
|  | not next to A/P genes | 163225 | 6% |
| **High PGP windows** | anywhere | 1319 | 14% |
|  | next to A/P genes | 132 (10%) | 51% |
|  | not next to A/P genes | 1187 | 10% |
