# Supplementary material for: Quantitative Analysis of the Drosophila Segmentation Regulatory Network Using Pattern Generating Potentials
Source: PLoS Biol. 2010 Aug 17;8(8):e1000456. doi: 10.1371/journal.pbio.1000456 (PMC2923081; doi:10.1371/journal.pbio.1000456)
Supplement: Text S1 — Supplementary notes. (Notes 1, 2, 3, 4, 5, 6, 7, 8, 9, 10.) (0.12 MB PDF) [file pbio.1000456.s024.pdf]

Text S1: Supplementary Notes

Note 1. CIC binding sites characterized by Bacterial 1-Hybrid technology [1].

CCATTCA

CCATTCA

CCATTCA

CCATTCA

CCATTCA

CCATTGA

CCATTGA

CCATTGA

CCATTGA

CCATTGA

CCATTGA

CCATTGA

CCATTGA

TCATTCA

TCATTCA

TCATTCA

TCATTCA

TCATTCA

TCATTGA

TCATTGA

TCCTTCA

CCCTTGC

GCACTCA

Note 2. In a given predicted expression profile, corresponding to a particular endogenous expression profile, let "APE" (**a**verage **p**rediction under **e**xpression) denote the average of the predicted expression values in bins that fall within domains of endogenous gene expression. Also let APNE (**a**verage **p**rediction under **n**on-**e**xpression) denote the average of predicted expression values in bins outside these domains of endogenous expression. The following figure shows the PGP score (equation 2 in text) as a function of APE and APNE. Note that the PGP score increases for higher values of APE (across each row), decreases for higher values of APNE (down each column), and decreases with identical increases in both APE and APNE (down the diagonals). All of these are intuitive and desirable properties of the PGP score.

average prediction under non-expression	Average prediction under expression										
	0	0.1	0.2	0.3	0.4	0.5	0.6	0.7	0.8	0.9	1
0	0.50	0.55	0.60	0.65	0.70	0.75	0.80	0.85	0.90	0.95	1.00
0.1	0.35	0.40	0.45	0.50	0.55	0.60	0.65	0.70	0.75	0.80	0.85
0.2	0.20	0.25	0.30	0.35	0.40	0.45	0.50	0.55	0.60	0.65	0.70
0.3	0.05	0.10	0.15	0.20	0.25	0.30	0.35	0.40	0.45	0.50	0.55
0.4	-0.10	-0.05	0.00	0.05	0.10	0.15	0.20	0.25	0.30	0.35	0.40
0.5	-0.25	-0.20	-0.15	-0.10	-0.05	0.00	0.05	0.10	0.15	0.20	0.25
0.6	-0.40	-0.35	-0.30	-0.25	-0.20	-0.15	-0.10	-0.05	0.00	0.05	0.10
0.7	-0.55	-0.50	-0.45	-0.40	-0.35	-0.30	-0.25	-0.20	-0.15	-0.10	-0.05
0.8	-0.70	-0.65	-0.60	-0.55	-0.50	-0.45	-0.40	-0.35	-0.30	-0.25	-0.20
0.9	-0.85	-0.80	-0.75	-0.70	-0.65	-0.60	-0.55	-0.50	-0.45	-0.40	-0.35
1	-1.00	-0.95	-0.90	-0.85	-0.80	-0.75	-0.70	-0.65	-0.60	-0.55	-0.50

However, since we are not interested in predictions that have APNE greater than or equal to APE, we updated the PGP score of equation 2 in text as follows:

$$Piecewise\ PGP = \begin{cases} PGP & APE > APNE \\ -0.5 & otherwise \end{cases}$$

Note 3. The control region of each gene is defined to be from minimum of its nearby upstream gene and 10 Kbp to minimum of its nearby downstream gene and 10kb, including the gene region itself. The only exceptions are the control regions of hairy and runt genes which are extended a further 5kb and 6kb either side respectively to include the known CRMs in their control region.

Note 4. The *gt* gene has two expression domains (bins ~15-40 and bins ~70-80), neither of which is recapitulated by the *gt_-6* CRM, which has an anterior terminal expression (bins 0-15). Since the PGP score is designed to find CRMs whose predicted expression profile matches endogenous gene expression, it fails to find the *gt_-6* CRM.

Note 5. Out of 10 TFs of interest, only BCD, KR and TorRE have reported target genes at 60% BLS confidence level with 181, 89 and 3 target genes respectively. Most of these three factors' target genes are outside of the AP gene set (i.e. in total, there are only 24 targets in AP gene set). The number of target genes predicted for these three factors (by the PGP method) are 44, 38 and 25 (for CIC) of which only 11 targets are shared with the BLS predictions [2]. This discrepancy might suggest that most of the A/P genes' functional transcription factor binding sites are located outside of 2kb promoter region used for the BLS analysis.

Note 6. Measures of prediction accuracy.
RMSE is the root of mean sum of square error [3] between the predicted and actual values.
Akaike Information Criterion (AIC) captures the goodness of fit and the complexity of a model as a single measure which can be used in model selection [4].
Pearson Correlation coefficient (CC) measures the linear dependence between two random variables [5], here the actual and predicted expression.

Note 7. Discretizing the expression profiles of the "FlyExpress" dataset. Starting with relative intensity values at each of 100 positions (bins) along the axis, obtained as described in Methods, we discretized the expression values by setting a threshold at 0.5 standard deviation above the axis-wide mean. The resultant binary profiles were manually inspected to obtain the best one for each gene.

Note 8. Multi Species Stubb. The MS_STUBB score of a window, for a given motif, is the Brownian Motion-based average of STUBB scores of that motif for the given window and its orthologs from 10 other *Drosophila* species. The MS_STUBB score of a window, for a given set of motifs, is the average of the motif specific MS_STUBB scores, over all motifs. MS_CBust refers to a run of Cluster Buster on *D. melanogaster* sequences where positions with Phastcons score below 0.9 have been masked, as suggested previously [6]. All programs were run with the following 10 transcription factors: BCD, CAD, HB, KNI, KR, GT, HKB, TLL, FKH, CIC), and with default settings for other parameters. To assess the performance of the PGP method, we used results from a cross-validation, where all known CRMs corresponding to a gene were left out to create the training data in each "fold".

Note 9. CIC and TorRE motif comparison. TorRE and CIC motifs were compared to each other using the relative entropy score, and an empirical p-value of this score was estimated using 10000 permutations of the longer motif.

Note 10. "False Positive" CRMs set. This dataset consists of eight experimentally tested sequences that contain a cluster of binding sites for A/P factors, but do not drive any detectable expression in the embryo. These are nub_+5, pdm2_+3, pdm2_+5 and pdm2_+8 from [7] and PCE8008, PCE8021, PCE8023 and PCE8007 from [8]. Additional bona fide CRMs from [8] are not considered because their neighboring genes are not A/P patterned, which implies that those non-CRMs will not even receive a score under our PGP scheme.

References

1. **Noyes, M.B., et al.,** ***A systematic characterization of factors that regulate Drosophila segmentation via a bacterial one-hybrid system.*** **Nucleic Acids Res, 2008. 36(8): p. 2547-60.**
2. **Kheradpour, P., et al.,** ***Reliable prediction of regulator targets using 12 Drosophila genomes.*** **Genome Res, 2007. 17(12): p. 1919-31.**
3. **Lehmann, E.L. and G. Casella,** ***Theory of Point Estimation (Springer Texts in Statistics)*****. 2003: Springer.**
4. **Akaike, H.,** ***A new look at the statistical model identification.*** **Automatic Control, IEEE Transactions on, 2003. 19(6): p. 716-723.**
5. **Rodgers, J. and A. Nicewander,** ***Thirteen Ways to Look at the Correlation Coefficient.*** **The American Statistician, 1988. 42(1): p. 59-66.**
6. **Aerts, S., et al.,** ***Fine-tuning enhancer models to predict transcriptional targets across multiple genomes.*** **PLoS One, 2007. 2(11): p. e1115.**
7. **Schroeder, M.D., et al.,** ***Transcriptional control in the segmentation gene network of Drosophila.*** **PLoS Biol, 2004. 2(9): p. E271.**
8. **Berman, B.P., et al.,** ***Computational identification of developmental enhancers: conservation and function of transcription factor binding-site clusters in Drosophila melanogaster and Drosophila pseudoobscura.*** **Genome Biol, 2004. 5(9): p. R61.**
