# Supplementary material for: Quantitative Analysis of the Drosophila Segmentation Regulatory Network Using Pattern Generating Potentials
Source: PLoS Biol. 2010 Aug 17;8(8):e1000456. doi: 10.1371/journal.pbio.1000456 (PMC2923081; doi:10.1371/journal.pbio.1000456)
Supplement: Text S2 — Supplementary methods. (0.11 MB PDF) [file pbio.1000456.s025.pdf]

Text S2: Supplementary Methods

June 28, 2010

1 Upward-Downward Algorithm

We consider the motif score as a random variable evolving according to the Brownian motion process along the branches of a phylogenetic tree. Using the Markov assumption, we can apply the upward-downward algorithm to efficiently compute the conditional distribution at unobserved node (internal nodes of the tree) given the observed values [?]. The upward-downward algorithm is analogous to the forward-backward algorithm associated with probability calculations of Hidden Markov Models [?]. In this section we describe the general notations for upward-downward algorithm as described in [?].

- X_i : random variable representing trait (observation) at node i
- x_i : value of trait (observation) at node i
- $C(i)$: children of node i
- $\pi(i)$: parent of node i
- $T(i)$: subtree rooted at node i
- t_i: branch length between node i and its immediate parent
- O_1: all observed traits in the tree
- $O_i = \{x_n | n \in T(i) \text{ and } C(n) = 0\}$: all observed traits in subtree rooted at node i
- $O_{i \backslash j} = \{x_n | n \in T(i) \text{ and } n \notin T(j) \text{ and } C(n) = 0\}$: all observed traits in subtree rooted at node i but not at node j
- δ_{m,x_i}

1.1 General Algorithm

To get the probavility distribution of an unobserved node, we first need to calculate the upward and downward probabilities. Next, we explain how to compute these two probabilities and then how to combine them to get the required distribution.

1.1.1 Generalized Upward Step

The upward probability of a node is defined as the probability of a subtree rooted at that node for any of its given observed value. For simplicity, we use the following notations:

- $\beta_i(m) = \Pr(O_i|X_i = m)$
- $\beta_{i,\pi(i)}(m) = \Pr(O_i|X_{\pi(i)} = m)$
- $\beta_{\pi(i)\setminus i}(m) = \Pr(O_{\pi(i)\setminus i}|X_{\pi(i)} = m)$

By initializing the values of $\beta_i(m) = \delta_{m,x_i}$ at the observed leaf nodes, we can work up the tree for each node $\pi(i)$ at the next level to calculate the values of interest as following:

1. $\beta_{i,\pi(i)}(m) = \begin{cases} \Pr(m \to x_i|t_i)\beta_i(x_i) & \text{if } C(i) = 0, \\ \sum_{m'} \Pr(m \to m'|t_i)\beta_i(m') & \text{otherwise,} \end{cases}$

   where $Pr(m \to m'|t_i)$ is the probability of value m' evolves to m in time t_i and will be explained later according to Brownian motion process.

2. $\beta_{\pi(i)}(m) = \prod_{j \in C(\pi(i))} \beta_{j,\pi(i)}(m)$

3. $\beta_{\pi(i)\setminus i}(m) = \frac{\beta_{\pi(i)}(m)}{\beta_{i,\pi(i)}(m)} = \beta_{i',\pi(i)}(m)$, where i' is sibling of i

1.1.2 Generalized Downward Step

The downward probability of a node is defined as the the probability of the observations outside the subtree rooted at the node for each possible value of the subtree root. For simpilicity, we use the following notation:

- $\alpha_i(m) = \Pr(X_i = m, O_{1\setminus i})$

By initializing the root of the phylogenetic tree with a prior probability distribution, $\alpha_1(m) = \Pr(X_1 = m) = \text{Prior}$, we can work down the tree to calculate the value of $\alpha_i(m)$ for all nodes in the next level of the tree as following:

- $\alpha_i(m) = \sum_{m'} \alpha_{\pi(i)}(m') \Pr(m' \to m|t_i) \beta_{\pi(i)\setminus i}(m')$
  where $Pr(m \to m'|t_i)$ is the probability of value m' evolves to m in time t_i and will be explained later according to Brownian motion process.

1.1.3 Marginals

Once the upward and the downward calculations are completed, we can compute the joint probabilities of all observations and values of each node as follow:

$$\Pr(X_i = m, O_1) = \alpha_i(m)\beta_i(m)$$

Finally, we calculate the conditional probability of the values at each node given the observations at all of the leaves.

$$\Pr(X_i = m|O_1) = \frac{\alpha_i(m)\beta_i(m)}{\sum_{m'} \alpha_i(m')\beta_i(m')}$$

1.2 Brownian Algorithm

By modeling the evolution of the random variable with Brownian motion, we can directly and simply compute and represent the continuous distributions at each node in the phylogenetic tree. We introduce a new variable, σ^2_{bm}, the variance in displacement per unit time for the Brownian motion process. A distribution of a random variable x subject to Brownian motion, with mean μ, and variance σ^2 (represented with the notation $\mathrm{N}(x, \mu, \sigma^2)$, have the Gaussian probability distribution function:

$$f(x) = \frac{1}{\sqrt{2\pi\sigma^2}} \exp^{\frac{-(x-\mu)^2}{2\sigma^2}}$$

1.2.1 Product of Two Gaussians

An important identity in the upward-downward calculations is that the product of two Gaussian distributions is Gaussian. In our notation:

- $N(x;\mu_1,\sigma_1^2)N(x;\mu_2,\sigma_2^2) = N(\mu_1;\mu_2,\sigma_1^2+\sigma_2^2)N(x;\mu,\sigma^2)$

where $\sigma^2 = \frac{1}{\frac{1}{\sigma_1^2}+\frac{1}{\sigma_2^2}}$ and $\mu = \sigma^2\left(\frac{\mu_1}{\sigma_1^2}+\frac{\mu_2}{\sigma_2^2}\right)$

1.2.2 Brownian Upward Step

For the initialization of the upward step, we will still represent the distribution at each leaf l with:
Initialization step:
$\beta_l(m) = \delta_{m,x_l}$

The three distributions of interest are iteratively calculated for node.
Iteration step:

1. At the leaf l:
   $\beta_{l,\pi(l)}(m) \sim N(m, x_l, \sigma_{bm}^2 t_l)$

   At other internal (unobserved traits) nodes in the phylogenetic tree, i:

   $$\beta_{i,\pi(i)}(m) = \int_{m'} N(m';m,\sigma_{bm}^2 t_i)\beta_i(m')dm'$$

   Let $\beta_i(m') = c_i N(m';\mu_i,\sigma_i^2)$, then

   $$\begin{aligned}\beta_{i,\pi(i)}(m) &= c_i\int_{m'} N(m';m,\sigma_{bm}^2 t_i)N(m';\mu_i,\sigma_i^2)dm' \\ &= c_i\int_{m'} N(m;\mu_i,\sigma_{bm}^2 t_i+\sigma_i^2)N(m';\mu_n,\sigma_n^2)dm'\end{aligned}$$

   where $\sigma_n^2 = \frac{1}{\frac{1}{\sigma_{bm}^2 t_i}+\frac{1}{\sigma_i^2}}$ and $\mu_n = \sigma_n^2\left(\frac{m}{\sigma_{bm}^2 t_i}+\frac{\mu_i}{\sigma_i^2}\right)$.
   Therefore,

   $$\begin{aligned}\beta_{i,\pi(i)}(m) &= c_i N(m;\mu i,\sigma_{bm}^2 t_i+\sigma_i^2)\underbrace{\int_{m'} N(m';\mu_n,\sigma_n^2)dm'}_{=1} \\ &= c_i N(m;\mu_i,\sigma_{bm}^2 t_i+\sigma_i^2)\end{aligned}$$

2. $\beta_{\pi(i)}(m) = \beta_{i,\pi(i)}(m)\beta_{i',\pi(i')}(m)$, where i' is the sibling of i.

   Let $\beta_{i,\pi(i)}(m) \sim c_i N(m;\mu_i,\sigma_i^2)$ and
   $\beta_{i',\pi(i')}(m) \sim c_{i'} N(m;\mu_{i'},\sigma_{i'}^2)$, then

$$\begin{aligned}\beta_{\pi(i)}(m) &= c_i c_{i'} N(m;\mu_i,\sigma_i^2)N(m;\mu_{i'},\sigma_{i'}^2)\\ &= c_i c_{i'} N(\mu_i;\mu_{i'},\sigma_i^2+\sigma_i^2)N(m;\mu_n,\sigma_n^2)\end{aligned}$$

   where $\sigma_n^2 = \frac{1}{\frac{1}{\sigma_i^2}+\frac{1}{\sigma_{i'}^2}}$ and $\mu_n = \sigma_n^2\left(\frac{\mu_i}{\sigma_i^2}+\frac{\mu_{i'}}{\sigma_{i'}^2}\right)$.

3. $\beta_{\pi(i)\backslash i}(m) = \beta_{i,\pi(i)}(m)$, where i' is the sibling of i.

1.2.3 Brownian Downward Step

The downward probabilities of the root of the tree are initialized to a uniform prior probability distribution $\sim U[0,20]$.
Initialization step:
$\alpha_1(m) = \Pr(X_1 = m) \sim U[0,20]$

For all internal nodes, i:
Iteration step:

$$\alpha_i(m) = \int_{m'} \alpha_{\pi(i)}(m')\beta_{\pi(i)\backslash i}(m')N(m;m',\sigma_{bm}^2 t_i)dm'$$

Let $\alpha_{\pi(i)}(m') = c_1 N(m';\mu_1,\sigma_1^2)$, and
let $\beta_{\pi(i)\backslash i}(m') = c_2 N(m';\mu_2,\sigma_2^2)$, then

$$\begin{aligned}\alpha_i(m) &= c_1 c_2 \int_{m'} N(m';\mu_1,\sigma_1^2)N(m';\mu_2,\sigma_2^2)N(m;m',\sigma_{bm}^2 t_i)dm'\\ &= c_1 c_2 c_3 \int_{m'} N(m';\mu_n,\sigma_2^n)N(m;m',\sigma_{bm}^2 t_i)dm'\end{aligned}$$

where $\sigma_n^2 = \frac{1}{\frac{1}{\sigma_1^2}+\frac{1}{\sigma_2^2}}$, $\mu_n = \sigma_n^2\left(\frac{\mu_1}{\sigma_1^2}+\frac{\mu_2}{\sigma_2^2}\right)$, and and $c_3 = N(\mu_1;\mu_2,\sigma_1^2+\sigma_2^2)$.

Therefore,

$$
\begin{aligned}
\alpha_i(m) &= c_1c_2c_3 \int_{m'} N(m;\mu_n,\sigma_2^n+\sigma_{bm}^2 t_i) N(m';\mu_{n'},\sigma_{n'}^2) dm' \\
&= c_1c_2c_3 N(m;\mu_n,\sigma_2^n+\sigma_{bm}^2 t_i) \underbrace{\int_{m'} N(m';\mu_{n'},\sigma_{n'}^2) dm'}_{=1} \\
&= c_1c_2c_3 N(m;\mu_n,\sigma_2^n+\sigma_{bm}^2 t_i)
\end{aligned}
$$

1.2.4 Marginals and Conditional Expectation

After the completion of the upward-downward algorithm, we calculate the joint probabilities of all observations and values of each node, i.
$\Pr(X_i = m, O_1) = \alpha_i(m)\beta_i(m)$

Let $\alpha_i(m) = c_1 N(m;\mu_1,\sigma_1^2)$, and
let $\beta_i(m) = c_2 N(m;\mu_2,\sigma_2^2)$, then

$$
\begin{aligned}
Pr(X_i = m, O_1) &= c_1c_2 N(m;\mu_1,\sigma_1^2) N(m;\mu_2,\sigma_2^2) \\
&= c_1c_2 N(\mu_1;\mu_2,\sigma_1^2+\sigma_1^2) N(m;\mu_n,\sigma_n^2)
\end{aligned}
$$

where $\sigma_n^2 = \frac{1}{\frac{1}{\sigma_1^2}+\frac{1}{\sigma_2^2}}$ and $\mu_n = \sigma_n^2 \left(\frac{\mu_1}{\sigma_1^2} + \frac{\mu_2}{\sigma_2^2}\right)$

We compute the conditional probability of the values at each node given the observations at all of the leaves as:

$$
\begin{aligned}
\Pr(X_i = m | O_1) &= \frac{\alpha_i(m)\beta_i(m)}{\int_{m'} \alpha_i(m')\beta_i(m') dm'} \\
&= \frac{c_1c_2 N(\mu_1;\mu_2,\sigma_1^2+\sigma_1^2) N(m;\mu_n,\sigma_n^2)}{c_1c_2 N(\mu_1;\mu_2,\sigma_1^2+\sigma_1^2) \int_{m'} N(m;\mu_n,\sigma_n^2) dm'} \\
&= \frac{N(m;\mu_n,\sigma_n^2)}{\int_{m'} N(m;\mu_n,\sigma_n^2) dm'}
\end{aligned}
$$

Therefore, the expected value $E(X_i|O_1)$ of this conditional probability is μ_n. The expected value of each branch between node i and $\pi(i)$ in the tree, is defined as:

- $\frac{1}{2}[E(X_i|O_1) + E(X_{\pi(i)}|O_1)]$.

The temporal average of the entire phylogenetic tree is calculated as the sum of the expected values of each branch weighted by its branch length.

Temporal Average $= \sum_{i \in T(1)} \frac{t_i}{2}[E(X_i|O_1) + E(X_{\pi(i)}|O_1)]$

References

[1] O. Ronen et al (1995) Parameter Estimation of Dependence Tree Models Using the EM Algorithm. *IEEE SIGNAL PROCESSING LETTERS* **2** No. 8, pp. 157-159.

[2] L. R. Rabiner (2002) A tutorial on hidden markov models and selected applications in speech recognition. *Proceedings of the IEEE*, 77(2), pp. 257286.
